# Histo-MRI map study protocol: a prospective cohort study mapping MRI to histology for biomarker validation and prediction of prostate cancer

**DOI:** 10.1136/bmjopen-2021-059847

**Published:** 2022-04-08

**Authors:** Saurabh Singh, Manju Mathew, Thomy Mertzanidou, Shipra Suman, Joey Clemente, Adam Retter, Marianthi-Vasiliki Papoutsaki, Lorna Smith, Francesco Grussu, Veeru Kasivisvanathan, Alistair Grey, Eoin Dinneen, Greg Shaw, Martyn Carter, Dominic Patel, Caroline M Moore, David Atkinson, Eleftheria Panagiotaki, Aiman Haider, Alex Freeman, Daniel Alexander, Shonit Punwani

**Affiliations:** 1 Centre for Medical Imaging, University College London, London, UK; 2 Department of Pathology, University College London Hospitals NHS Foundation Trust, London, UK; 3 Centre for Medical Imaging Computing, Department of Computer Science, University College London, London, UK; 4 Radiomics Group, Vall d’Hebron Barcelona Hospital Campus, Barcelona, Spain; 5 Division Of Surgery and Interventional Sciences, University College London, London, UK; 6 Department of Urology, University College London Hospitals NHS Foundation Trust, London, UK; 7 Department of Urology, Barts Health NHS Trust, London, UK; 8 Faculty of the Built Environment, University College London, London, UK; 9 Department of Pathology, University College London Cancer Institute, London, UK

**Keywords:** Magnetic resonance imaging, Prostate disease, Pathology

## Abstract

**Introduction:**

Multiparametric MRI (mpMRI) is now widely used to risk stratify men with a suspicion of prostate cancer and identify suspicious regions for biopsy. However, the technique has modest specificity and a high false-positive rate, especially in men with mpMRI scored as indeterminate (3/5) or likely (4/5) to have clinically significant cancer (csPCa) (Gleason ≥3+4). Advanced MRI techniques have emerged which seek to improve this characterisation and could predict biopsy results non-invasively. Before these techniques are translated clinically, robust histological and clinical validation is required.

**Methods and analysis:**

This study aims to clinically validate two advanced MRI techniques in a prospectively recruited cohort of men suspected of prostate cancer. Histological analysis of men undergoing biopsy or prostatectomy will be used for biological validation of biomarkers derived from Vascular and Extracellular Restricted Diffusion for Cytometry in Tumours and Luminal Water imaging. In particular, prostatectomy specimens will be processed using three-dimension printed patient-specific moulds to allow for accurate MRI and histology mapping. The index tests will be compared with the histological reference standard to derive false positive rate and true positive rate for men with mpMRI scores which are indeterminate (3/5) or likely (4/5) to have clinically significant prostate cancer (csPCa). Histopathological validation from both biopsy and prostatectomy samples will provide the best ground truth in validating promising MRI techniques which could predict biopsy results and help avoid unnecessary biopsies in men suspected of prostate cancer.

**Ethics and dissemination:**

Ethical approval was granted by the London—Queen Square Research Ethics Committee (19/LO/1803) on 23 January 2020. Results from the study will be presented at conferences and submitted to peer-reviewed journals for publication. Results will also be available on ClinicalTrials.gov.

**Trial registration number:**

NCT04792138.

**Strengths and limitations of this study**

- A prospective cohort of men suspected of prostate cancer referred to two tertiary care centres in the UK representative of the population of interest.
- The reference standard to validate novel imaging biomarkers includes both targeted biopsy and matched whole-mount prostatectomy histology without participants deviating from standard clinical care.
- The patient-specific specimen handling protocol for prostatectomy which was developed at this institution allows for accurate matching of MRI and histology.
- This protocol will produce a rich imaging and histology dataset that can be used to train machine learning algorithms and validate novel imaging biomarkers.
- Novel imaging biomarkers will not influence clinical decisions, which is ethically sound for novel imaging biomarkers which have not been validated clinically.

## Introduction

The diagnosis of prostate cancer has been transformed by multiparametric MRI (mpMRI) which has become the first line investigation for men suspected to have prostate cancer in many countries.^1^ However, diagnosis still relies on invasive biopsy for confirmation. This diagnostic pathway has two main limitations (1) The specificity of mpMRI is modest (as low as 41%) and leads to unnecessary negative biopsies and (2) There is a sampling error associated with biopsy as it is can miss abnormalities identified on MRI.^2 3^

The poor specificity of mpMRI is due to several factors. Benign diseases such as inflammation and atrophy can mimic tumours or make tumours less conspicuous leading to indeterminate results when assessed by radiologists.^4–6^ The mpMRI study is assessed qualitatively rather than quantitatively to determine the presence of clinically significant cancer (csPCa) which causes interobserver variation and subjectivity. Both these factors can lead to a high false-positive rate in men who undergo biopsy after assessment of their mpMRI. Results from a recent trial at our institution showed that men undergoing biopsy with mpMRI scores of Likert 3 (indeterminate for csPCa) and Likert 4 (likely to have csPCa) had false-positive rates of 85% and 40%, respectively.^7^ However, when mpMRI is scored as highly likely to have csPCa (Likert 5/5), the percentage of false positives is low at 2%.^7^ Therefore, there is a clinical need for biomarkers to better stratify men with Likert 3 or Likert 4 mpMRI scores to reduce false positives without missing men with csPCa.

Advanced MRI techniques designed specifically for the prostate aim to improve cancer detection and characterisation by inferring microstructural information from the whole prostate non-invasively. Vascular, Extracellular and Restricted Diffusion for Cytometry in Tumours (VERDICT MRI) is a specifically designed diffusion technique based on prostate histology that derives estimates of histological parameters non-invasively.^8 9^ Technical validation and early biological validation have been achieved with results outperforming standard diffusion sequences.^10 11^ Luminal IndexImaging (LWI) is an advanced T2-based technique that has similarly been technically and biologically validated.^12^ Quantitative evaluation of both these techniques could assist radiologists to reduce false positives when mpMRIs are scored as Likert 3 or 4 on the likelihood of csPCa.

Histological validation from biopsy alone has limitations of only validating small regions of the prostate. In contrast, histological validation from prostatectomy specimens leads to a selection bias where men with abnormal prostates are selected rather than those men who may have indolent or benign diseases. Furthermore, the prostate can be sliced in a different axis in histopathology compared with imaging which causes imperfect matching and poor validation. A patient-specific specimen handling for prostatectomy specimens can overcome these limitations and allow better matching of MRI and whole-mount histopathology.^13^

In this study, both biopsy and MRI matched whole-mount histology from prostatectomy will be used for clinical validation of novel MRI techniques in a prospective cohort of men suspected of prostate cancer. In particular, the impact of index tests on the false positive rate in men who are scored Likert 3 or 4 and underwent biopsy or prostatectomy will be evaluated. The decision to biopsy or recommendation for prostatectomy will not be influenced by the novel MRI techniques being validated.

## Methods and analysis

### Study Design

**Figure 1:**
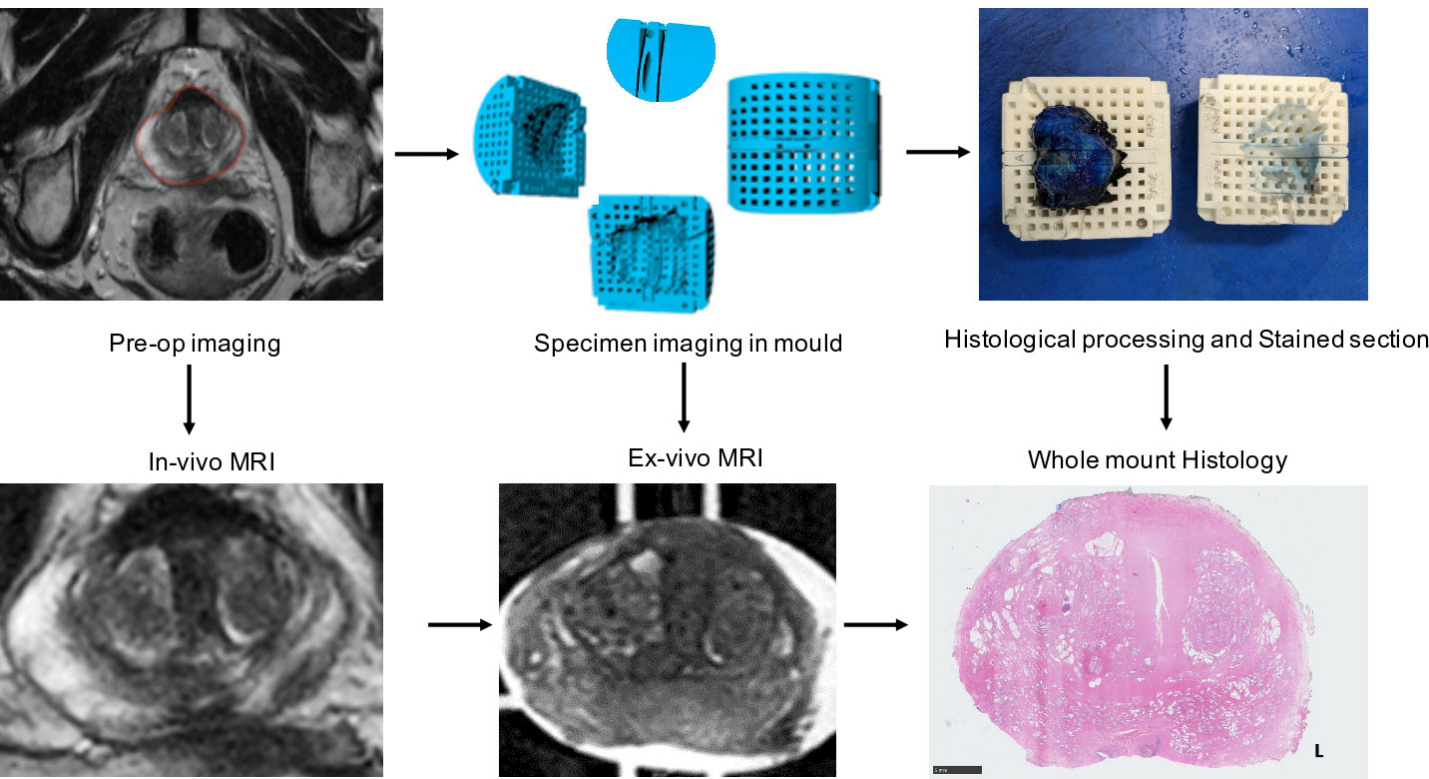
Protocol for matching MRI to Whole mount histology. The participant’s prostate is contoured by a radiologist on preoperative imaging (‘in vivo MRI’) slice by slice. Based on these contours, a mould specific to the participant’s prostate is 3D printed. The ‘reference slice’ is predefined based on the location of the tumour. After prostatectomy, the prostate is scanned in the mould (‘ex-vivo MRI’). The prostate is sectioned first at the predefined reference slice. The remainder of the prostate is then sliced as standard. Stained ‘whole Mount histology’ is then matched with ex vivo and in vivo imaging. 3D, three dimensions.

Histo-MRI is an observational, prospective, cohort study recruiting men with clinical suspicion of prostate cancer from two centres: University College London Hospital (UCLH) and Barts Health. The index tests (VERDICT MRI and LWI) and standard of care mpMRI will be performed at one centralised centre (UCLH). The study opened for recruitment in October 2020 and the anticipated study end date is October 2023. Participants will undergo the both tests before undergoing biopsy if indicated by the standard test. The index tests will not be used to select patients for biopsy. If a recruited participant is diagnosed with prostate cancer and chooses to undergo prostatectomy, the prostate specimen will undergo a specific specimen handling protocol designed to align histopathology to MRI (figure 1).

The primary objective is to assess whether the index tests can reduce false positives from mpMRI by 20% for men undergoing biopsy or biopsy and prostatectomy. Clinically significant prostate cancer (csPCa) for biopsies is defined as any single biopsy core containing Gleason grade 3+4 or above. For prostatectomy, csPCa is defined as the predominant grade of 3+4 or above in the same matched region on whole mount histology as the abnormality on MRI.

The secondary objectives will examine the proportions of true positive lesions from the index tests.

Exploratory analyses will determine the correlation of VERDICT and LWI parameters to histological parameters. For VERDCIT MRI: fractional intracellular fraction (FIC) to fractional histological intracellular component, fractional extracellular extravascular space to histological glandular and stromal component and fractional vascular volume (FVASC) to histological vascular component. For LWI, luminal water fraction (LWF) will be correlated to luminal space fraction.

Matched MR and histology images will be used as training data for machine learning algorithms. The algorithms will be used to solve a variety of regression problems where the input is MRI and the output is the histological features in each voxel or the full histological image appearance itself, building on previous work from our group.^14–16^

### Participants

Participants will be approached consecutively from Urology clinics at the point of referral from two centres: UCLH Foundation Trust, London, UK and Barts Health, London, UK. Inclusion and exclusion criteria are stated in box 1. Informed consent will be obtained on the day of the index test and participants will be given at least 24 hours before being consented to consider participation.

Box 1 **Inclusion, exclusion and withdrawal criteria**

**Patient inclusion criteria**

Biopsy naïve men with clinical suspicion of prostate cancer.

**Patient exclusion criteria**

Men unable to have an MRI scan or in whom artifactartefact would reduce quality of MRI.

Men unable to given informed consent.

Previous treatment of prostate cancer (surgery, radiotherapy, hormone treatment).

Previous biopsy.

**Withdrawal criteria**

Images inadequate for analysis.

### Index tests

The index tests of VERDICT MRI and LWI will be performed on a 3T MRI scanner (Achieva or Ingenia, Philips Healthcare, Best, Netherlands). Sequence parameters are detailed in tables 1 and 2. These will be additional to the clinical multiparametric sequence. Total scan time will be a maximum of 1 hour.

**Table 1:** Sequence parameters for Verdict MRI

Verdict MRI		
**MR scanner**	**Achieva (3T**)	**Ingenia (3T**)
Receive coil (s)	32 channel Cardiac coil	Body coils
Sequence	DWI SE EPI single shot	DWI SE EPI single shot
Field of view (mm)	220	220
No of slices	14	14
Slice thickness (mm)	5	5
Slice gap (mm)	0	0
phase encoding direction	AP	AP
Reconstructed matrix	176×176	176×176
Reconstructed pixel size (mm)	1.25	1.25
**b-values**	0.3000	0.3000
Repetition time (TR) range, actual (ms)	3349–10 000,2260	3349–10 000, 6292
Echo time (TE) (ms)	80	87
Water fat shift WFS(pix)/Bandwidth(Hz)	49.09/8.8	57.54/7.5
DELTA/delta (ms)	38.8/18.9	43.4/20.0
No of signal averages	6	6
**b-values**	0.2000	0.2000
TR range, actual (ms)	2000–10000, 3897	2000–10000, 6699
TE(ms)	67	75
WFS(pix)/BW(Hz)	49.09/8.8	57.55,7.5
DELTA/delta (ms)	32.3/12.4	37.4, 14.0
Number of signal averages	6	6
**b-values**	0.1500	0.1500
TR range, actual (ms)	2000–10000, 2398	2000–10000, 2967
TE(ms)	90	94
WFS(pix)/BW(Hz)	49.09/8.8	58.05, 7.5
DELTA/delta (ms)	43.8/23.9	46.9, 23.3
Number of signal averages	6	6
**b-values**	0.500	0.500
TR range, actual (ms)	2482–10000, 2482	2000–10000, 2229
TE(ms)	65	68
WFS(pix)/BW(Hz)	49.06/8.8	58.05, 7.5
DELTA/delta (ms)	31.3, 11.4	33.9, 10.3
Number of signal averages	6	6
**b-values**	0.90	0.90
TR range, actual (ms)	2482–10000, 2482	2000–10000, 2024
TE (ms)	50	54
WFS(pix)/BW(Hz)	49.09,8.8	57.54, 7.5
DELTA/delta (ms)	23.8/3.9	26.9, 3.5
No of signal averages	4	4
Acquisition time (minute: second)	10:95	17:41

DWI SE EPI, Diffusion Weighted Imaging Spin Echo Echo Planar Imaging.

**Table 2:** Sequence parameters for Luminal Index Imaging MRI

Luminal index imaging		
**MR scanner**	**Achieva**	**Ingenia**
Receive coil (s)	32 channel Cardiac coil	Body Coils
**Sequence**	TSE (multishot)	FSE
**FOV (mm**)	180	180
N of slices	19	19
**Slice thickness (mm**)	3.5	3.5
**Slice gap (mm**)	0.35	0.35
Phase encoding direction	Right Left	Right Left
Reconstructed matrix (read)	224×224	224×224
Reconstructed pixel size (mm x mm)	0.94×0.94	0.94×0.94
Echo times (ms)	31.25/62.5/93.8/125/156.3/187.5/218.8/250	31.25/62.5/93.8/125/156.3/187.5/218.8/250
Repetition time (ms)	Shortest (7676 ms)	Shortest (7676 ms)
No of echoes	8	8
Receive bandwidth	WFS 2.99 / (144.8.6 Hz/px)	WFS 2.99 / (144.8.6 Hz/px)
No of signal averages	1	1
Turbo factor	8	8
Acquisition time (minute: second)	06:44	05:39

FSE, Fast Spin Echo; TSE, Turbo Spin Echo; WFS, water fat shift.

The VERDICT MRI technique has been described in previous publications^11^; a summary is given below. VERDICT uses a pulse-gradient spin-echo sequence acquired with a 32-channel cardiac coil with b values of 90–3000 s/mm^2^ in 3 orthogonal directions. For b=90 s/mm^2^, the number of signal averages (NSA) was 4 and for other b values, the NSA was 6. The voxel size is 1.3×1.3×5 mm, matrix size=176×176. A b=0 s/mm^2^ image for every echo time (TE) is acquired to mitigate T2 dependence. After processing, estimates of intracellular volume fraction (FIC), extravascular extracellular volume fraction (EES), vascular volume fraction (FVASC) are generated by a previously described method.^17^

LWI comprises of a multiecho spinecho sequence with an echo spacing of 31.25 ms and repetition time (TR) of 8956 msec. The field of view (FOV) is 180×180 mm and acquired voxel size=0.9×0.9×3.5 mm with a scan duration of 5 min 50 s. LWF values for lesions identified on mpMRI will be calculated after data processing by a previously described method.^7^

### Data analysis

Reporting of clinical mpMRI will follow standard of care and be reported by Uro-radiologists based at UCLH on an ordinal Likert scale (1–5): 1—tumour highly unlikely, 2—tumour unlikely, 3—equivocal, 4—tumour likely and 5—tumour highly likely. If the mpMRI study is reported as Likert three or four and the patient is offered a biopsy, a radiologist (blinded to biopsy results/histopathology) will use the pictorial report to derive index test quantitative parameters: FIC for VERDICT MRI and LWF from LWI. Thresholds of index test parameters based on previous work from the INNOVATE trial will be applied to determine whether a lesion is positive or negative.^7^ This will be compared with a histological reference standard allowing assessment of false positive rate, sensitivity and specificity.

### Reference standard

**Figure 2:**
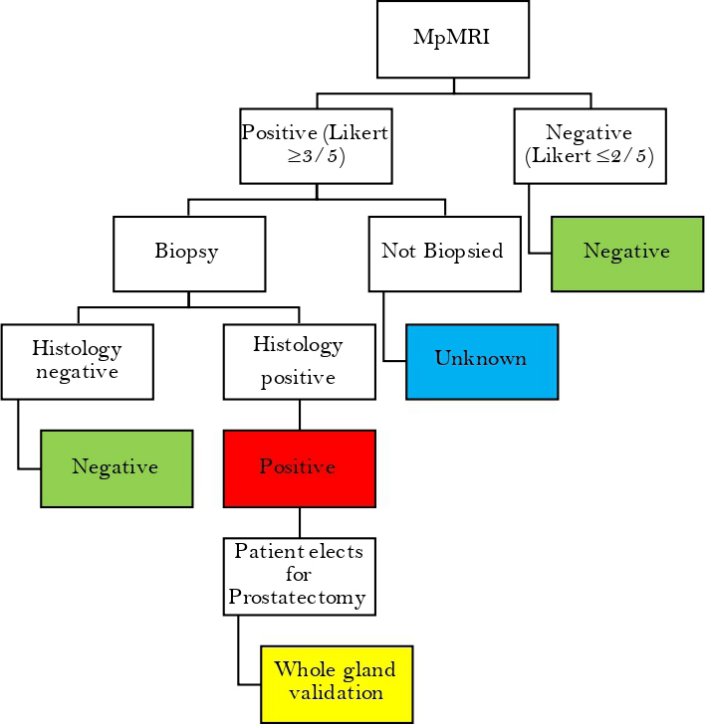
Reference standard flow chart reference standard derived from multiparametric MRI (mpMRI) and histology for index tests. Histology refers to either a positive biopsy core in a targeted lesion or positive lesion on matched MRI and whole mount histology from prostatectomy. Histology from prostatectomy supersedes targeted biopsy.

The reference standard for those men who undergo biopsy and/or prostatectomy will be histopathological diagnosis (figure 2). Abnormal regions identified by clinical mpMRI will be targeted at biopsy. Men who elect to have prostatectomy after positive biopsy will be processed by a published specimen handling protocol developed at our institution.^13^ Histology from biopsy and prostatectomy specimens will be assessed by two histopathologists blinded to MRI findings. Gleason grade for targeted biopsy and matched prostatectomy whole block section will be analysed and ascribed a Gleason score on consensus. Assessors of the reference standard will be blinded to MRI results. If there is a disagreement in histological assessment for a participant who undergoes both targeted biopsy and prostatectomy, the prostatectomy lesion grading will supersede.

For patients who undergo prostatectomy, in order to match MRI slices to histopathology, a patient-specific specimen handling protocol is to be used^13^ (figure 1). This protocol is needed because in standard histological processing there are changes in orientation after cutting and sampling which are difficult to account for when matching standard whole mount histology to MRI (figure 1). We create a personalised three-dimension printed mould for men undergoing prostatectomy using their preoperative MRI images (T2W imaging) in order to allow for predictable sectioning.

After surgical removal of the prostate the specimen will be positioned in the mould and scanned in a 3T MRI scanner. This will facilitate the matching between preoperative MRI and histology.

The mould defines a reference slice which has two cutting planes spaced 5 mm apart. A twin bladed knife is used to cut a 5 mm slice predefined from the mould. The rest of the prostate is then sliced at 5 mm thickness as per standard laboratory protocol. Whole mount sections are then processed for H&E staining. Additional immunohistochemical staining for vascular and stromal structures will be performed to aide in mapping with the index tests.

The matched whole mount histology slice will be assessed and used to determine whether an MRI lesion was positive (≥Gleason 3+4) or negative (≤Gleason 3+3 or negative).

### Statistical analyses

A sample size of 128 subjects achieves a 90% power to detect a difference of 20% in the proportion of false positives between the index tests (0.45) and standard test (0.65). This calculation uses a two-sided Pearson χ^2^ test with a significance level of 0.05 and confidence level of 95%. We anticipate a biopsy rate of 57% in the cohort and of those biopsied, 75% to be scored Likert 3 or 4, based on results from the recent INNOVATE trial at our institution.^7^ Therefore, a sample size of 300 will provide sufficient power, with an estimated 171 men predicted to have biopsy and of these men, 128 to have a Likert score of 3 or 4.

Approximately 800 patients undergo prostatectomy per year at UCLH and 150 are referred from Barts Health National Health Service(NHS) trust. We estimate in a sample size of 300, approximately 50 patients will elect to have a prostatectomy based on clinical experience and recruitment in other studies carried out at our institution.

The difference in proportion of false positives and true positives for the index test will be compared with the standard test (mpMRI) using a χ^2^ test. Correlation coefficients will be used to determine the correlation between index test parameters and histological measures.

### Patient and public involvement

There has been no formal involvement of the patient group or public in the design of this protocol. However, participant feedback from recent research studies such as INNOVATE^7^ has informed the study design. For instance, participants will be offered research scans on the same appointment date as their hospital appointment for convenience.

## Discussion

Histology remains the gold standard of prostate cancer diagnosis and therefore represents the best reference standard for novel MRI techniques. This observational study aims to test the predictive capabilities of novel MRI techniques without compromising on standard clinical care. Furthermore, matched MRI and histological data will provide a rich data set for training machine learning algorithms.

This study design has some limitations. Biopsy decisions will not be influenced by the index tests therefore we cannot determine their true sensitivity and specificity. However, at this stage of biomarker development, prospective validation of thresholds derived from a previous study is required before biopsy decisions could be determined by the index tests.^7^ Given the high negative predictive value of mpMRI,^18 19^ it would also lead to unnecessary morbidity if patients with negative mpMRI are biopsied. The inherent sampling error with biopsies is also a limitation of this study in men who do not undergo prostatectomy. However, if only men undergoing prostatectomy were selected in this study, histological validation will be limited by spectrum bias where more aggressive tumours are selected.

The results of this study may not be generalisable to other centres. The two index tests have been optimised on scanners from the same vendor. Formal reproducibility studies are required to assess whether the index tests perform as well on different systems before multicentre trials can be performed.^20^

## Conclusion

Histo-MRI is a prospective, observational study, which aims to test the potential value of novel MRI techniques in diagnosis of significant prostate cancer in men that undergo biopsy following mpMRI. The results of this study will provide histological validation for novel MRI techniques and produce a rich dataset which can be used to train machine learning algorithms for prostate cancer diagnosis and prognosis.

### Ethics and dissemination

The study is sponsored by UCLH. The UCL/UCLH joint research office maintains responsibility for monitoring of Good Clinical Practice in the study. Ethical approval for the study was granted by the London—Queen Square Research Ethics Committee (19/LO/1803) on 23 January 2020. Study results will be presented at conferences and submitted to peer-reviewed journals.

## Supplementary Material

Reviewer comments

Author's manuscript
