# Supplementary material for: Histo-MRI map study protocol: a prospective cohort study mapping MRI to histology for biomarker validation and prediction of prostate cancer
Source: BMJ Open. 2022 Apr 8;12(4):e059847. doi: 10.1136/bmjopen-2021-059847 (PMC8995953; doi:10.1136/bmjopen-2021-059847)
Supplement: Author's manuscript [file bmjopen-2021-059847.draft_revisions.pdf]

BMJ Open is committed to open peer review. As part of this commitment we make the peer review history of every article we publish publicly available.

When an article is published we post the peer reviewers' comments and the authors' responses online. We also post the versions of the paper that were used during peer review. These are the versions that the peer review comments apply to.

The versions of the paper that follow are the versions that were submitted during the peer review process. They are not the versions of record or the final published versions. They should not be cited or distributed as the published version of this manuscript.

BMJ Open is an open access journal and the full, final, typeset and author-corrected version of record of the manuscript is available on our site with no access controls, subscription charges or pay-per-view fees (http://bmjopen.bmj.com).

If you have any questions on BMJ Open's open peer review process please email info.bmjopen@bmj.com

BMJ Open

Histo-MRI map: A prospective cohort study mapping MRI to histology for biomarker validation and prediction of prostate cancer

Journal:	*BMJ Open*
Manuscript ID	bmjopen-2021-059847
Article Type:	Protocol
Date Submitted by the Author:	17-Dec-2021
Complete List of Authors:	Singh, Saurabh; University College London,<br>Mathew, Manju; University College London, Centre for Medical Imaging; University College London Hospitals NHS Foundation Trust, Department of Pathology<br>Mertzanidou, Thomy; University College London, Centre for Medical Imaging Computing, Department of Computer Science<br>Suman, Shipra; University College London, Centre for Medical Imaging; University College London, Centre for Medical Imaging Computing, Department of Computer Science<br>Clemente, Joey; University College London, Centre for Medical Imaging<br>Retter, Adam; University College London, Centre for Medical Imaging<br>Papoutsaki, Marianthi-Vasiliki; University College London<br>Smith, Lorna; University College London, Centre for Medical Imaging<br>Grussu, Francesco; University College London, Centre for Medical Imaging Computing, Department of Computer Science; Vall d'Hebron Barcelona Hospital Campus<br>Kasivisvanathan, Veeru; University College London, Division Of Surgery and Interventional Sciences<br>Grey, Alistair; University College London Hospitals NHS Foundation Trust, Department of Urology<br>Dinneen, Eoin; University College London Medical School, Department of Surgical and Interventional Sciences<br>Shaw, Greg; University College Hospital London, Department of Urology<br>Carter, Martyn; University College London, Faculty of the Built Environment<br>Patel, Dominic; University College London Cancer Institute, Department of Pathology<br>Moore, Caroline; University College London, Division of Surgery and Interventional Sciences<br>Atkinson, David; University College London, Centre for Medical Imaging<br>Panagiotaki, Eleftheria; University College London, Centre for Medical Imaging Computing, Department of Computer Science<br>Haider, Aiman; University College London Hospitals NHS Foundation Trust, Department of Histopathology<br>Freeman, Alex; University College Hospital London, Department of Histopathology<br>Alexander, Daniel; University College London, Department of Computer Science<br>Punwani, Shonit; University College London Medical School, Centre for

	Medical Imaging
Keywords:	Magnetic resonance imaging < RADIOLOGY & IMAGING, Prostate disease < UROLOGY, Pathology < NATURAL SCIENCE DISCIPLINES

SCHOLARONE™
Manuscripts

Histo-MRI map: A prospective cohort study mapping MRI to histology for biomarker validation and prediction of prostate cancer

Saurabh Singh[1*†], Manju Mathew[1,2†], Thomy Mertzanidou[3], Shipra Suman[1,3], Joey Clemente[1], Adam Retter[1], Marianthi-Vasiliki Papoutsaki[1], Lorna Smith[1], Francesco Grussu[3,4], Veeru Kasivisvanathan[5,6] Alistair Grey[6,7], Eoin Dinneen[6], Greg Shaw[6,7], Martyn Carter[8], Dominic Patel[9], Caroline Moore[5,6], David Atkinson[1], Eleftheria Panagiotaki[3], Aiman Haider[2], Alex Freeman[2], Daniel Alexander[3], Shonit Punwani[1]

1. Centre for Medical Imaging, Division of Medicine, University College London, London, UK
2. Department of Pathology, University College London Hospitals NHS Foundation Trust, London, UK
3. Centre for Medical Imaging Computing, Department of Computer Science, University College London, UK
4. Radiomics Group, Vall d'Hebron Institute of Oncology, Vall d'Hebron Barcelona Hospital Campus, Barcelona, Spain
5. Division of Surgery & Interventional Science, University College London, London, UK
6. Department of Urology, University College London Hospitals NHS Foundation Trust, London, UK
7. Department of Urology, Barts Health NHS Foundation Trust, London, UK
8. Faculty of the Built Environment, The Bartlett School of Architecture, University College London, London, UK
9. Department of Pathology, University College London Cancer Institute, London, UK

* Corresponding author. Centre of Medical Imaging, University College London, Charles Bell House, 43-45 Foley Street, London W1W 7TS, UK.

saurabh.singh@nhs.net, Tel +44 0207 679 8156

For peer review only - http://bmjopen.bmj.com/site/about/guidelines.xhtml

† Equal contributors

Abstract

Introduction:

Multiparametric magnetic resonance imaging (mpMRI) is now widely used to risk stratify men with a suspicion of prostate cancer and identify suspicious regions for biopsy. However, the technique has modest specificity and a high false-positive rate, especially in men with mpMRI scored as indeterminate (3/5) or likely (4/5) to have clinically significant cancer (Gleason ≥3+4). Advanced MRI techniques have emerged which seek to improve this characterisation and could predict biopsy results non-invasively. Before these techniques are translated clinically, robust histological and clinical validation is required.

Methods and Analysis:

This study aims to clinically validate two advanced MRI techniques in a prospectively recruited cohort of men suspected of prostate cancer. Histological analysis of men undergoing biopsy or prostatectomy will be used for biological validation of biomarkers derived from VERDICT (Vascular and Extracellular Restricted Diffusion for Cytometry in Tumours) and Luminal Water imaging (LWI). In particular, prostatectomy specimens will be processed using 3-D printed patient-specific moulds to allow for accurate MRI and histology mapping. The index tests will be compared to the histological reference standard to derive false positive rate and true positive rate for men with mpMRI scores which are indeterminate (3/5) or likely (4/5) to have clinically significant prostate cancer (csPCa). Histopathological validation from both biopsy and prostatectomy samples will provide the best ground truth in validating promising MRI techniques which could predict biopsy results and help avoid unnecessary biopsies in men suspected of prostate cancer.

Ethics and Dissemination

For peer review only - http://bmjopen.bmj.com/site/about/guidelines.xhtml

Ethical approval was granted by the London – Queen Square Research Ethics Committee (19/LO/1803) on 23rd January 2020. Results from the study will be presented at conferences and submitted to peer-reviewed journals for publication. Results will also be available on ClinicalTrials.gov.

Article Summary

Strengths and limitations of this study

- A prospective cohort of men suspected of prostate cancer referred to two tertiary care centres in the UK representative of the population of interest.
- The reference standard to validate novel imaging biomarkers includes both targeted biopsy and matched whole-mount prostatectomy histology without participants deviating from standard clinical care.
- The patient-specific specimen handling protocol for prostatectomy which was developed at this institution allows for accurate matching of MRI and Histology.
- This protocol will produce a rich imaging and histology dataset that can be used to train machine learning algorithms and validate novel imaging biomarkers.
- Novel imaging biomarkers will not influence clinical decisions, which is ethically sound for novel imaging biomarkers which have not been validated clinically.

Trial registration: Histo-MRI is registered on ClinicalTrials.gov; reference NCT04792138.

Keywords: Prostate cancer, Magnetic Resonance Imaging, VERDICT MRI, Luminal Index Imaging, Prostatectomy, RALP, Machine learning

Word Count: 2252

For peer review only

1. Introduction

The diagnosis of prostate cancer has been transformed by multiparametric MRI (mpMRI) which has become the first line investigation for men suspected to have prostate cancer in many countries[1]. However, diagnosis still relies on invasive biopsy for confirmation. This diagnostic pathway has two main limitations i) The specificity of mpMRI is modest (as low as 40%) and leads to unnecessary negative biopsies ii) There is a sampling error associated with biopsy as it is can miss abnormalities identified on MRI[2][3].

The poor specificity of mpMRI is due to several factors. Benign diseases such as inflammation and atrophy can mimic tumours or make tumours less conspicuous leading to indeterminate results when assessed by radiologists [4–6]. The mpMRI study is assessed qualitatively rather than quantitatively to determine the presence of clinically significant cancer (csPCa) which causes inter-observer variation and subjectivity. Both these factors can lead to a high false-positive rate in men who undergo biopsy after assessment of their mpMRI. Results from a recent trial at our institution showed that men undergoing biopsy with mpMRI scores of Likert 3 (indeterminate for csPCa) and Likert 4 (likely to have csPCa) had false-positive rates of 85% and 40% respectively [7]. However, when mpMRI is scored as highly likely to have csPCa (Likert 5/5), the percentage of false positives is low at 2% [7]. Therefore, there is a clinical need for biomarkers to better stratify men with Likert 3 or Likert 4 mpMRI scores to reduce false positives without missing men with csPCa.

Advanced MRI techniques designed specifically for the prostate aim to improve cancer detection and characterisation by inferring microstructural information from the whole

For peer review only

For peer review only - http://bmjopen.bmj.com/site/about/guidelines.xhtml

prostate non-invasively. Vascular, Extracellular and Restricted Diffusion for Cytometry in Tumours (VERDICT MRI) is a specifically designed diffusion technique based on prostate histology that derives estimates of histological parameters non-invasively[8, 9]. Technical validation and early biological validation have been achieved with results outperforming standard diffusion sequences[10, 11]. Luminal index imaging (LWI) is an advanced T2 based technique that has similarly been technically and biologically validated[12]. Quantitative evaluation of both these techniques could assist radiologists to reduce false positives when mpMRIs are scored as Likert 3 or 4 on the likelihood of csPCa.

Histological validation from biopsy alone has limitations of only validating small regions of the prostate. In contrast, histological validation from prostatectomy specimens leads to a selection bias where men with abnormal prostates are selected rather than those men who may have indolent or benign diseases. Furthermore, the prostate can be sliced in a different axis in histopathology compared to imaging which causes imperfect matching and poor validation. A patient-specific specimen handling for prostatectomy specimens can overcome these limitations and allow better matching of MRI and whole-mount histopathology[13]

In this study, both biopsy and MRI matched whole-mount histology from prostatectomy will be used for clinical validation of novel MRI techniques in a prospective cohort of men suspected of prostate cancer. In particular, the impact of index tests on the false positive rate in men who are scored Likert 3 or 4 and underwent biopsy or prostatectomy will be evaluated. The decision to biopsy or recommendation for prostatectomy will not be influenced by the novel MRI techniques being validated.

For peer review only

2. Methods and analysis

2.1. Study Design

Histo-MRI is an observational, prospective, cohort study recruiting men with clinical suspicion of prostate cancer from two centres: University College London Hospital (UCLH) and Barts Health. The index tests (VERDICT MRI and LWI) and standard of care mpMRI will be performed at one centralised centre (UCLH). Participants will undergo the both tests before undergoing biopsy if indicated by the standard test. The index tests will not be used to select patients for biopsy. If a recruited participant is diagnosed with prostate cancer and chooses to undergo prostatectomy, the prostate specimen will undergo a specific specimen handling protocol designed to align histopathology to MRI (Figure 1).

The primary objective is to assess whether the index tests can reduce false positives from mpMRI by 20% for men undergoing biopsy or biopsy and prostatectomy. Clinically significant prostate cancer (csPCa) for biopsies is defined as any single biopsy core containing Gleason grade 3+4 or above. For prostatectomy, csPCa is defined as the predominant grade of 3+4 or above in the same matched region on whole mount histology as the abnormality on MRI.

The secondary objectives will examine the proportions of true positive lesions from the index tests.

Exploratory analyses will determine the correlation of VERDICT and LWI parameters to histological parameters. For VERDCIT MRI: fractional intracellular fraction (FIC) to

For peer review only - http://bmjopen.bmj.com/site/about/guidelines.xhtml

fractional histological intracellular component, fractional extracellular extravascular space (FEES) to histological glandular and stromal component and fractional vascular volume (FVASC) to histological vascular component. For LWI, luminal water fraction (LWF) will be correlated to luminal space fraction.

Matched MR and histology images will be used as training data for machine learning algorithms. The algorithms will be used to solve a variety of regression problems where the input is MRI and the output is the histological features in each voxel or the full histological image appearance itself, building on previous work from our group[14–16].

2.2. Participants

Participants will be approached consecutively from Urology clinics at the point of referral from two centres: University College London Hospital Foundation Trust, London, UK and Barts Health, London, UK. Inclusion and exclusion criteria are stated in Table 1. Informed Consent will be obtained on the day of the index test and participants will be given at least 24 hours before being consented to consider participation.

2.3. Index tests

The index tests of VERDICT MRI and LWI will be performed on a 3T MRI scanner (Achieva or Ingenia, Philips Healthcare, Best, Netherlands). Sequence parameters are detailed in Table 2 and 3. These will be additional to the clinical multiparametric sequence. Total scan time will be a maximum of 1 hour.

For peer review only

The VERDICT MRI technique has been described in previous publications[11]; a summary is given below. VERDICT uses a pulse-gradient spin-echo sequence acquired with a 32-channel cardiac coil with b values of 90-3000 s/mm^2 in 3 orthogonal directions. For b=90 s/mm^2, the number of signal averages (NSA) was 4 and for other b values, the NSA was 6. The voxel size is 1.3mm x 1.3mm x 5 mm, matrix size =176 x 176. A b=0 s/mm^2 image for every echo time (TE) is acquired to mitigate T2 dependence. After processing, estimates of intracellular volume fraction (FIC), extravascular extracellular volume fraction (EES), vascular volume fraction (FVASC) are generated by a previously described method[17].

LWI comprises of a multi-echo spin-echo sequence with an echo spacing of 31.25 msec and repetition time (TR) of 8956 msec. The field of view (FOV) is 180 × 180 mm and acquired voxel size = 0.9 mm × 0.9 mm × 3.5 mm with a scan duration of 5 minutes 50 seconds. LWF values for lesions identified on mpMRI will be calculated after data processing by a previously described method[7].

2.4. Data analysis

Reporting of clinical mpMRI will follow standard of care and be reported by Uro-radiologists based at UCLH on an ordinal Likert scale (1 to 5): 1- tumour highly unlikely, 2- tumour unlikely, 3- equivocal, 4- tumour likely and 5- tumour highly likely. If the mpMRI study is reported as Likert 3 or 4 and the patient is offered a biopsy, a radiologist (blinded to biopsy results/histopathology) will use the pictorial report to derive index test quantitative parameters: FIC for VERDICT MRI and LWF from LWI. Thresholds of index test parameters based on previous work from the INNOVATE trial will be applied to determine whether a lesion is positive or negative[7]. This will be

For peer review only - http://bmjopen.bmj.com/site/about/guidelines.xhtml

compared to a histological reference standard allowing assessment of false positive rate, sensitivity and specificity.

2.5. Reference Standard

The reference standard for those men who undergo biopsy and/or prostatectomy will be histopathological diagnosis (Figure 2). Abnormal regions identified by clinical mpMRI will be targeted at biopsy. Men who elect to have prostatectomy after positive biopsy will be processed by a published specimen handling protocol developed at our institution[13]. Histology from biopsy and prostatectomy specimens will be assessed by two histopathologists blinded to MRI findings. Gleason grade for targeted biopsy and matched prostatectomy whole block section will be analysed and ascribed a Gleason score on consensus. Assessors of the reference standard will be blinded to MRI results. If there is a disagreement in histological assessment for a participant who undergoes both targeted biopsy and prostatectomy, the prostatectomy lesion grading will supersede.

For patients that undergo prostatectomy, in order to match MRI slices to histopathology, a patient specific specimen handling protocol is to be used[13] (Figure 1). This protocol is needed because in standard histological processing there are changes in orientation after cutting and sampling which are difficult to account for when matching standard whole mount histology to MRI (Figure 1). We create a personalised 3-D printed mould for men undergoing prostatectomy using their pre-operative MRI images (T2W imaging) in order to allow for predictable sectioning.

For peer review only - http://bmjopen.bmj.com/site/about/guidelines.xhtml

After surgical removal of the prostate the specimen will be positioned in the mould and scanned in a 3T MRI scanner. This will facilitate the matching between pre-operative MRI and histology.

The mould defines a reference slice which has two cutting planes spaced 5 mm apart. A twin bladed knife is used to cut a 5 mm slice pre-defined from the mould. The rest of the prostate is then sliced at 5 mm thickness as per standard laboratory protocol. Whole mount sections are then processed for haematoxylin and eosin staining. Additional immunohistochemical staining for vascular and stromal structures will be performed to aide in mapping with the index tests.

The matched whole mount histology slice will be assessed and used to determine whether an MRI lesion was positive (≥ Gleason 3+4) or negative (≤Gleason 3+3 or negative).

2.6. Statistical Analyses

A sample size of 128 subjects achieves a 90% power to detect a difference of 20% in the proportion of false positives between the index tests (0.45) and standard test (0.65). This calculation uses a two-sided Pearson Chi-Square test with a significance level of 0.05 and confidence level of 95%. We anticipate a biopsy rate of 57% in the cohort and of those biopsied, 75% to be scored Likert 3 or 4, based on results from the recent INNOVATE trial at our institution[7]. Therefore, a sample size of 300 will provide sufficient power, with an estimated 171 men predicted to have biopsy and of these men, 128 to have a Likert score of 3 or 4.

For peer review only - http://bmjopen.bmj.com/site/about/guidelines.xhtml

Approximately 800 patients undergo prostatectomy per year at UCLH and 150 are referred from Barts Health NHS trust. We estimate in a sample size of 300, approximately 50 patients will elect to have a prostatectomy based on clinical experience and recruitment in other studies carried out at our institution.

The difference in proportion of false positives and true positives for the index test will be compared to the standard test (mpMRI) using a Chi-square test. Correlation coefficients will be used to determine the correlation between index test parameters and histological measures.

2.7. Patient and public involvement

There has been no formal involvement of the patient group or public in the design of this protocol. However, participant feedback from recent research studies such as INNOVATE[7] has informed the study design. For instance, participants will be offered research scans on the same appointment date as their hospital appointment for convenience.

3. Discussion

Histology remains the gold standard of prostate cancer diagnosis and therefore represents the best reference standard for novel MRI techniques. This observational study aims to test the predictive capabilities of novel MRI techniques without compromising on standard clinical care. Furthermore, matched MRI and histological data will provide a rich data set for training machine learning algorithms.

For peer review only

This study design has some limitations. Biopsy decisions will not be influenced by the index tests therefore we cannot determine their true sensitivity and specificity. However, at this stage of biomarker development, prospective validation of thresholds derived from a previous study is required before biopsy decisions could be determined by the index tests [7]. Given the high negative predictive value of mpMRI[18, 19], it would also lead to unnecessary morbidity if patients with negative mpMRI are biopsied. The inherent sampling error with biopsies is also a limitation of this study in men who do not undergo prostatectomy. However, if only men undergoing prostatectomy were selected in this study, histological validation will be limited by spectrum bias where more aggressive tumours are selected.

The results of this study may not be generalisable to other centres. The two index tests have been optimised on scanners from the same vendor. Formal reproducibility studies are required to assess whether the index tests perform as well on different systems before multi-centre trials can be performed[20].

Conclusion

Histo-MRI is a prospective, observational study, which aims to test the potential value of novel MRI techniques in diagnosis of significant prostate cancer in men that undergo biopsy following mpMRI. The results of this study will provide histological validation for novel MRI techniques and produce a rich dataset which can be used to train machine learning algorithms for prostate cancer diagnosis and prognosis.

4. Ethics and Dissemination

For peer review only - http://bmjopen.bmj.com/site/about/guidelines.xhtml

The study is sponsored by University College London. The UCL/UCLH joint research office maintains responsibility for monitoring of Good Clinical Practice in the study. Ethical approval for the study was granted by the London – Queen Square Research Ethics Committee (19/LO/1803) on 23rd January 2020. Study results will be presented at conferences and submitted to peer reviewed journals.

List of abbreviations

MpMRI – multiparametric magnetic resonance imaging

VERDICT - Vascular and Extracellular Restricted Diffusion for Cytometry in Tumours

LWI – Luminal Index imaging

UCLH – University College London Hospital

UCL – University College London

FIC – Fractional Intracellular Volume

FEES – Fractional Extracellular extravascular space

FVASC – Fractional vascular space

LWF – Luminal water fraction

csPCa – Clinically significant prostate cancer

TE – Echo time

TR – Time to repetition

FOV – Field of view

For peer review only - http://bmjopen.bmj.com/site/about/guidelines.xhtml

- Contribution statement
  - Study concept and initial design: DA, SP, TM, EP
  - Study design and statistical analysis: SS, FG, TM, AH, AF, DA, SP
  - Data acquisition and analysis: SS, MM, TM, ShS, JC, AR, VP, LS, VK, AG, ED, GS, MC, DP, CM, EP, AH, AF.
  - All authors read and approved the final manuscript.
- Competing interests statement
  - The work of Baris Kanber, Shonit Punwani and Daniel Alexander is supported by the National Institute for Health Research University College London Hospitals Biomedical Research Centre.
  - Veeru Kasivisvanathan is an Academic Clinical Lecturer funded by the United Kingdom National Institute for Health Research (NIHR). The views expressed in this publication are those of the author(s) and not necessarily those of the NHS, the National Institute for Health Research or the Department of Health.
  - CMM is supported by an NIHR Research Professorship, and has additional study funding from the Medical Research Council, Cancer Research UK, PCUK, Movember, and the European Association of Urology Research Board.

For peer review only - http://bmjopen.bmj.com/site/about/guidelines.xhtml

- FG is currently supported by PREdICT, an investigator-initiated study at the Vall d'Hebron Hospital campus (Barcelona, Spain), funded by AstraZeneca.

- Funding statement
    - This work is supported by Engineering and Physical Sciences Research Council (EPSRC), grant reference (EP/R006032/1) and EP/M020533/1.

- Data Sharing Statement
    - All data relevant to the study are included in the article.

REFERENCES

1. Recommendations | Prostate cancer: diagnosis and management | Guidance | NICE.

2. Ahmed HU, El-Shater Bosaily A, Brown LC, Gabe R, Kaplan R, Parmar MK, et al. Diagnostic accuracy of multi-parametric MRI and TRUS biopsy in prostate cancer (PROMIS): a paired validating confirmatory study. Lancet. 2017;389:815–22.

3. Kasivisvanathan V, Rannikko AS, Borghi M, Panebianco V, Mynderse LA, Vaarala MH, et al. MRI-targeted or standard biopsy for prostate-cancer diagnosis. N Engl J Med. 2018;378:1767–77.

4. Kitzing YX, Prando A, Varol C, Karczmar GS, Maclean F, Oto A. Benign conditions that mimic prostate carcinoma: MR imaging features with histopathologic correlation. Radiographics. 2016;36:162–75. doi:10.1148/rg.2016150030.

5. Osses DF, Arsov C, Schimmöller L, Schoots IG, van Leenders GJLH, Esposito I, et al. Equivocal pi-rads three lesions on prostate magnetic resonance imaging: Risk stratification strategies to avoid mri-targeted biopsies. J Pers Med. 2020;10:1–13. doi:10.3390/jpm10040270.

6. Schoots IG. MRI in early prostate cancer detection: How to manage indeterminate or equivocal PI-RADS 3 lesions? Translational Andrology and Urology. 2018;7:70–82.

For peer review only - http://bmjopen.bmj.com/site/about/guidelines.xhtml

doi:10.21037/tau.2017.12.31.

7. Pye H, Singh S, Norris JM, Carmona Echeverria LM, Stavrinides V, Grey A, et al. Evaluation of PSA and PSA Density in a Multiparametric Magnetic Resonance Imaging-Directed Diagnostic Pathway for Suspected Prostate Cancer: The INNOVATE Trial. Cancers (Basel). 2021;13:1985. doi:10.3390/cancers13081985.

8. Panagiotaki E, Chan RW, Dikaios N, Ahmed HU, O'Callaghan J, Freeman A, et al. Microstructural characterization of normal and malignant human prostate tissue with vascular, extracellular, and restricted diffusion for cytometry in tumours magnetic resonance imaging. Invest Radiol. 2015;50:218–27.

9. Panagiotaki E, Walker-Samuel S, Siow B, Johnson SP, Rajkumar V, Pedley RB, et al. Noninvasive quantification of solid tumor microstructure using VERDICT MRI. Cancer Res. 2014;74:1902–12. doi:10.1158/0008-5472.CAN-13-2511.

10. Bailey C, Bourne RM, Siow B, Johnston EW, Brizmohun Appayya M, Pye H, et al. VERDICT MRI validation in fresh and fixed prostate specimens using patient-specific moulds for histological and MR alignment. NMR Biomed. 2019;32.

11. Johnston EW, Bonet-Carne E, Ferizi U, Yvernault B, Pye H, Patel D, et al. VERDICT MRI for prostate cancer: Intracellular volume fraction versus apparent diffusion coefficient. Radiology. 2019;291:391–7.

12. Devine W, Giganti F, Johnston EW, Sidhu HS, Panagiotaki E, Punwani S, et al. Simplified Luminal Water Imaging for the Detection of Prostate Cancer From Multiecho T_2 MR Images. J Magn Reson Imaging. 2019;50:910–7. doi:10.1002/jmri.26608.

13. Bourne RM, Bailey C, Johnston EW, Pye H, Heavey S, Whitaker H, et al. Apparatus for histological validation of in vivo and ex vivo magnetic resonance imaging of the human prostate. Front Oncol. 2017;7 MAR:47. doi:10.3389/fonc.2017.00047.

14. Jacobs JG, Panagiotaki E, Alexander DC. Gleason grading of prostate tumours with max-margin conditional random fields. Lect Notes Comput Sci (including Subser Lect

For peer review only

Notes Artif Intell Lect Notes Bioinformatics). 2014;8679:85–92. doi:10.1007/978-3-319-10581-9_11.

15. Tanno R, Worrall DE, Ghosh A, Kaden E, Sotiropoulos SN, Criminisi A, et al. Bayesian Image Quality Transfer with CNNs: Exploring Uncertainty in dMRI Super-Resolution. Lect Notes Comput Sci (including Subser Lect Notes Artif Intell Lect Notes Bioinformatics). 2017;10433 LNCS:611–9. http://arxiv.org/abs/1705.00664. Accessed 11 Feb 2021.

16. Alexander DC, Zikic D, Zhang J, Zhang H, Criminisi A. Image quality transfer via random forest regression: Applications in diffusion MRI. In: Lecture Notes in Computer Science (including subseries Lecture Notes in Artificial Intelligence and Lecture Notes in Bioinformatics). Springer Verlag; 2014. p. 225–32. doi:10.1007/978-3-319-10443-0_29.

17. Panagiotaki E, Walker-Samuel S, Siow B, Johnson SP, Rajkumar V, Pedley RB, et al. Noninvasive quantification of solid tumor microstructure using VERDICT MRI. Cancer Res. 2014;74:1902–12. doi:10.1158/0008-5472.CAN-13-2511.

18. Sathianathen NJ, Omer A, Harriss E, Davies L, Kasivisvanathan V, Punwani S, et al. Negative Predictive Value of Multiparametric Magnetic Resonance Imaging in the Detection of Clinically Significant Prostate Cancer in the Prostate Imaging Reporting and Data System Era: A Systematic Review and Meta-analysis. European Urology. 2020;78:402–14. doi:10.1016/j.eururo.2020.03.048.

19. Itatani R, Namimoto T, Atsuji S, Katahira K, Morishita S, Kitani K, et al. Negative predictive value of multiparametric MRI for prostate cancer detection: outcome of 5-year follow-up in men with negative findings on initial MRI studies. Eur J Radiol. 2014;83:1740–5. doi:10.1016/j.ejrad.2014.06.026.

20. O'Connor JPB, Aboagye EO, Adams JE, Aerts HJWL, Barrington SF, Beer AJ, et al. Imaging biomarker roadmap for cancer studies. Nat Rev Clin Oncol. 2017;14:169–86. doi:10.1038/nrclinonc.2016.162.

For peer review only

Figure Legends

Figure 1 Protocol for matching MRI to Whole mount histology.

The participant's prostate is contoured by a radiologist on pre-operative imaging ('in-vivo MRI') slice by slice. Based on these contours a mould specific to the participant's prostate is 3-D printed. The 'reference slice' is pre-defined based on the location of the tumour. After prostatectomy, the prostate is scanned in the mould ('ex-vivo MRI'). The prostate is sectioned first at the pre-defined reference slice. The remainder of the prostate is then sliced as standard. Stained 'whole mount histology' is then matched with ex-vivo and in-vivo imaging.

Figure 2 Reference standard flow chart

Reference standard derived from multi-parametric (mpMRI) and histology for index tests. Histology refers to either a positive biopsy core in a targeted lesion or positive lesion on matched MRI and whole mount histology from prostatectomy. Histology from prostatectomy supersedes targeted biopsy.

For peer review only

Table 1 – Inclusion, exclusion and withdrawal criteria

**Patient Inclusion Criteria**
Biopsy naïve men with clinical suspicion of prostate cancer
**Patient Exclusion Criteria**
Men unable to have an MRI scan or in whom artifact would reduce quality of MRI
Men unable to given informed consent
Previous treatment of prostate cancer (surgery, radiotherapy, hormone treatment)
Previous biopsy
Withdrawal Criteria
Images inadequate for analysis

For peer review only

Table 2 – Sequence parameters for VERDICT MRI

VERDICT MRI		
**MR scanner**	**Achieva (3T)**	**Ingenia (3T)**
Receive coil (s)	32 channel Cardiac coil	Body coils
Sequence	DWI SE EPI single shot	DWI SE EPI single shot
Field of View (mm)	220	220
Number of slices	14	14
Slice thickness (mm)	5	5
Slice gap (mm)	0	0
phase encoding direction	AP	AP
Reconstructed matrix	176 x 176	176 x 176
Reconstructed pixel size (mm)	1.25	1.25
**b-values**	0,3000	0,3000
Repetition time (TR) range, actual (ms)	3349-10000,2260	3349-10000, 6292
Echo time (TE) (ms)	80	87
Water fat shift WFS(pix)/Bandwidth(Hz)	49.09/8.8	57.54/7.5
DELTA/delta (ms)	38.8/18.9	43.4/20.0
Number of signal averages	6	6
**b-values**	0, 2000	0, 2000
TR range, actual (ms)	2000-10000, 3897	2000-10000, 6699
TE(ms)	67	75
WFS(pix)/BW(Hz)	49.09/8.8	57.55,7.5
DELTA/delta (ms)	32.3/12.4	37.4, 14.0
Number of signal averages	6	6
**b-values**	0,1500	0, 1500
TR range, actual (ms)	2000-10000, 2398	2000-10000, 2967

For peer review only - http://bmjopen.bmj.com/site/about/guidelines.xhtml

TE(ms)	90	94
WFS(pix)/BW(Hz)	49.09/8.8	58.05, 7.5
DELTA/delta (ms)	43.8/23.9	46.9, 23.3
Number of signal averages	6	6
**b-values**	0, 500	0, 500
TR range, actual (ms)	2482-10000, 2482	2000-10000, 2229
TE(ms)	65	68
WFS(pix)/BW(Hz)	49.06/8.8	58.05, 7.5
DELTA/delta (ms)	31.3, 11.4	33.9, 10.3
Number of signal averages	6	6
**b-values**	0, 90	0, 90
TR range, actual (ms)	2482-10000, 2482	2000-10000, 2024
TE(ms)	50	54
WFS(pix)/BW(Hz)	49.09,8.8	57.54, 7.5
DELTA/delta (ms)	23.8/3.9	26.9, 3.5
Number of signal averages	4	4
Acquisition Time (minutes: seconds)	10:95	17:41

Table 3 – Sequence parameters for Luminal Index Imaging MRI

**Luminal Index Imaging**		
**MR scanner**	**Achieva**	**Ingenia**
Receive coil (s)	32 channel Cardiac coil	Body Coils
**Sequence**	TSE (multishot)	FSE
**FOV (mm)**	180	180
Number of slices	19	19
**Slice thickness (mm)**	3.5	3.5
**Slice gap (mm)**	0.35	0.35
Phase Encoding direction	Right Left	Right Left
Reconstructed matrix (read)	224 x 224	224 x 224
Reconstructed pixel size (mm x mm)	0.94 x 0.94	0.94 x 0.94
Echo times (ms)	31.25/ 62.5/ 93.8/ 125/ 156.3/ 187.5/ 218.8/ 250	31.25/ 62.5/ 93.8/ 125/ 156.3/ 187.5/ 218.8/ 250
Repetition time (ms)	Shortest (7676ms)	Shortest (7676ms)
Number of echoes	8	8
Receive bandwidth	WFS 2.99 / (144.8.6Hz/px)	WFS 2.99 / (144.8.6Hz/px)
Number of signal averages	1	1
Turbo factor	8	8
Acquisition time (minutes: seconds)	06:44	05:39

For peer review only - http://bmjopen.bmj.com/site/about/guidelines.xhtml

For peer review only

For peer review only - http://bmjopen.bmj.com/site/about/guidelines.xhtml

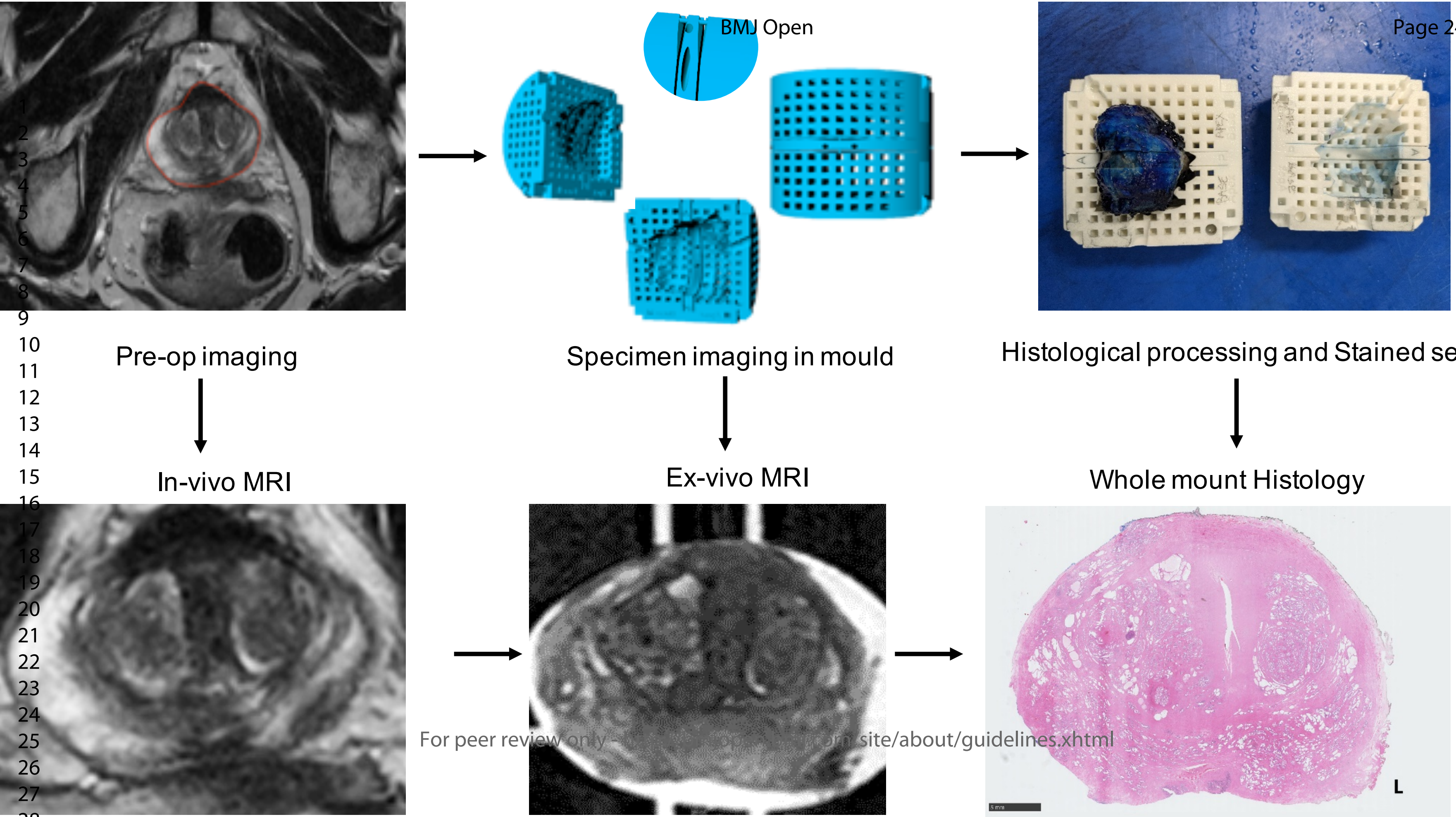
Pre-op imaging
Specimen imaging in mould
Histological processing and Stained section
In-vivo MRI
Ex-vivo MRI
Whole mount Histology
L

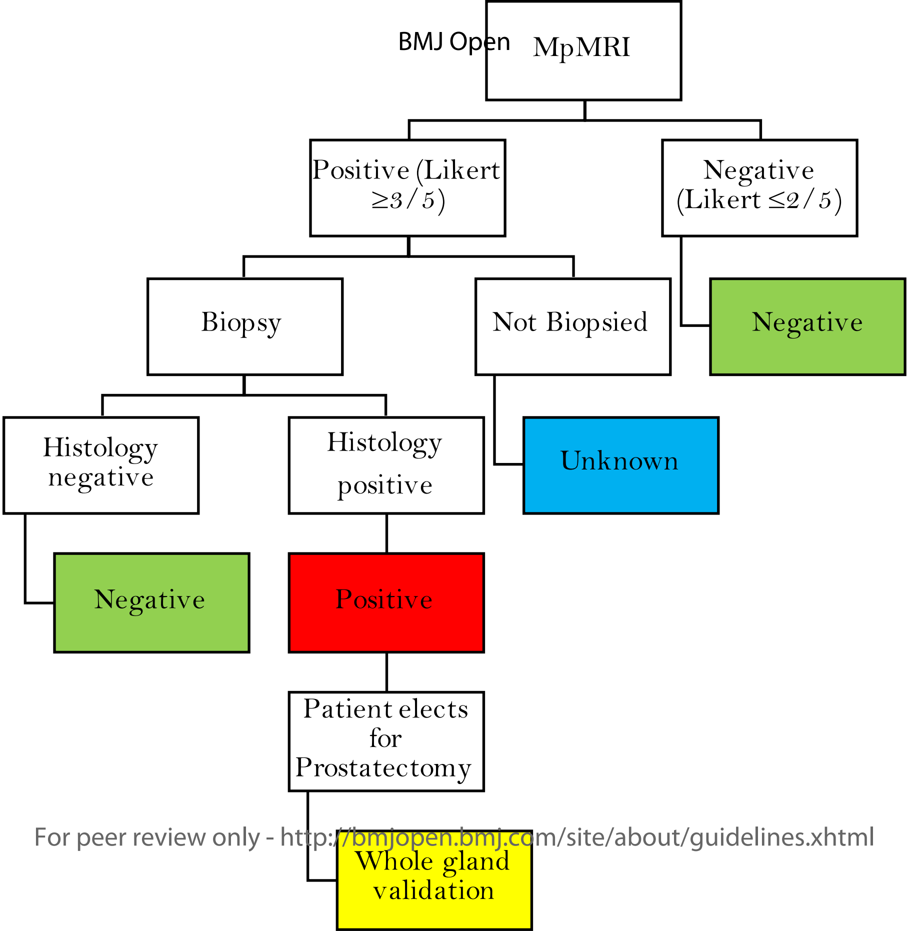

For peer review only - http://bmjopen.bmj.com/site/about/guidelines.xhtml

Section & Topic	No	Item	Reported on page #
**TITLE OR ABSTRACT**			
	**1**	Identification as a study of diagnostic accuracy using at least one measure of accuracy (such as sensitivity, specificity, predictive values, or AUC)	1
**ABSTRACT**			
	**2**	Structured summary of study design, methods, results, and conclusions (for specific guidance, see STARD for Abstracts)	2
**INTRODUCTION**			
	**3**	Scientific and clinical background, including the intended use and clinical role of the index test	4
	**4**	Study objectives and hypotheses	4
**METHODS**			
Study design	**5**	Whether data collection was planned before the index test and reference standard were performed (prospective study) or after (retrospective study)	6
Participants	**6**	Eligibility criteria	7
	**7**	On what basis potentially eligible participants were identified (such as symptoms, results from previous tests, inclusion in registry)	7
	**8**	Where and when potentially eligible participants were identified (setting, location and dates)	7
	**9**	Whether participants formed a consecutive, random or convenience series	7
Test methods	**10a**	Index test, in sufficient detail to allow replication	6
	**10b**	Reference standard, in sufficient detail to allow replication	7, 8
	**11**	Rationale for choosing the reference standard (if alternatives exist)	7
	**12a**	Definition of and rationale for test positivity cut-offs or result categories of the index test, distinguishing pre-specified from exploratory	7, 8
	**12b**	Definition of and rationale for test positivity cut-offs or result categories of the reference standard, distinguishing pre-specified from exploratory	7, 8
	**13a**	Whether clinical information and reference standard results were available to the performers/readers of the index test	7, 8
	**13b**	Whether clinical information and index test results were available to the assessors of the reference standard	7, 8
Analysis	**14**	Methods for estimating or comparing measures of diagnostic accuracy	8
	**15**	How indeterminate index test or reference standard results were handled	8
	**16**	How missing data on the index test and reference standard were handled	8
	**17**	Any analyses of variability in diagnostic accuracy, distinguishing pre-specified from exploratory	9, 10
	**18**	Intended sample size and how it was determined	10
**RESULTS**			
Participants	**19**	Flow of participants, using a diagram	N/A
	**20**	Baseline demographic and clinical characteristics of participants	N/A
	**21a**	Distribution of severity of disease in those with the target condition	N/A
	**21b**	Distribution of alternative diagnoses in those without the target condition	N/A
	**22**	Time interval and any clinical interventions between index test and reference standard	N/A
Test results	**23**	Cross tabulation of the index test results (or their distribution) by the results of the reference standard	N/A
	**24**	Estimates of diagnostic accuracy and their precision (such as 95% confidence intervals)	N/A
	**25**	Any adverse events from performing the index test or the reference standard	N/A
**DISCUSSION**			
	**26**	Study limitations, including sources of potential bias, statistical uncertainty, and generalisability	11
	**27**	Implications for practice, including the intended use and clinical role of the index test	NA
**OTHER INFORMATION**			
	**28**	Registration number and name of registry	3
	**29**	Where the full study protocol can be accessed	3
	**30**	Sources of funding and other support; role of funders	21

For peer review only - http://bmjopen.bmj.com/site/about/guidelines.xhtml

For peer review only

STARD 2015

AIM

STARD stands for "Standards for Reporting Diagnostic accuracy studies". This list of items was developed to contribute to the completeness and transparency of reporting of diagnostic accuracy studies. Authors can use the list to write informative study reports. Editors and peer-reviewers can use it to evaluate whether the information has been included in manuscripts submitted for publication.

EXPLANATION

A **diagnostic accuracy study** evaluates the ability of one or more medical tests to correctly classify study participants as having a **target condition.** This can be a disease, a disease stage, response or benefit from therapy, or an event or condition in the future. A medical test can be an imaging procedure, a laboratory test, elements from history and physical examination, a combination of these, or any other method for collecting information about the current health status of a patient.

The test whose accuracy is evaluated is called **index test.** A study can evaluate the accuracy of one or more index tests. Evaluating the ability of a medical test to correctly classify patients is typically done by comparing the distribution of the index test results with those of the **reference standard**. The reference standard is the best available method for establishing the presence or absence of the target condition. An accuracy study can rely on one or more reference standards.

If test results are categorized as either positive or negative, the cross tabulation of the index test results against those of the reference standard can be used to estimate the **sensitivity** of the index test (the proportion of participants *with* the target condition who have a positive index test), and its **specificity** (the proportion *without* the target condition who have a negative index test). From this cross tabulation (sometimes referred to as the contingency or "2x2" table), several other accuracy statistics can be estimated, such as the positive and negative **predictive values** of the test. Confidence intervals around estimates of accuracy can then be calculated to quantify the statistical **precision** of the measurements.

If the index test results can take more than two values, categorization of test results as positive or negative requires a **test positivity cut-off**. When multiple such cut-offs can be defined, authors can report a receiver operating characteristic (ROC) curve which graphically represents the combination of sensitivity and specificity for each possible test positivity cut-off. The **area under the ROC curve** informs in a single numerical value about the overall diagnostic accuracy of the index test.

The **intended use** of a medical test can be diagnosis, screening, staging, monitoring, surveillance, prediction or prognosis. The **clinical role** of a test explains its position relative to existing tests in the clinical pathway. A replacement test, for example, replaces an existing test. A triage test is used before an existing test; an add-on test is used after an existing test.

Besides diagnostic accuracy, several other outcomes and statistics may be relevant in the evaluation of medical tests. Medical tests can also be used to classify patients for purposes other than diagnosis, such as staging or prognosis. The STARD list was not explicitly developed for these other outcomes, statistics, and study types, although most STARD items would still apply.

DEVELOPMENT

This STARD list was released in 2015. The 30 items were identified by an international expert group of methodologists, researchers, and editors. The guiding principle in the development of STARD was to select items that, when reported, would help readers to judge the potential for bias in the study, to appraise the applicability of the study findings and the validity of conclusions and recommendations. The list represents an update of the first version, which was published in 2003.

More information can be found on http://www.equator-network.org/reporting-guidelines/stard.

For peer review only

CC BY SA

BMJ Open

Histo-MRI map study protocol: A prospective cohort study mapping MRI to histology for biomarker validation and prediction of prostate cancer

Journal:	*BMJ Open*
Manuscript ID	bmjopen-2021-059847.R1
Article Type:	Protocol
Date Submitted by the Author:	02-Feb-2022

Complete List of Authors:	Singh, Saurabh; University College London,<br>Mathew, Manju; University College London, Centre for Medical Imaging; University College London Hospitals NHS Foundation Trust, Department of Pathology<br>Mertzanidou, Thomy; University College London, Centre for Medical Imaging Computing, Department of Computer Science<br>Suman, Shipra; University College London, Centre for Medical Imaging; University College London, Centre for Medical Imaging Computing, Department of Computer Science<br>Clemente, Joey; University College London, Centre for Medical Imaging<br>Retter, Adam; University College London, Centre for Medical Imaging<br>Papoutsaki, Marianthi-Vasiliki; University College London<br>Smith, Lorna; University College London, Centre for Medical Imaging<br>Grussu, Francesco; University College London, Centre for Medical Imaging Computing, Department of Computer Science; Vall d'Hebron Barcelona Hospital Campus<br>Kasivisvanathan, Veeru; University College London, Division Of Surgery and Interventional Sciences<br>Grey, Alistair; University College London Hospitals NHS Foundation Trust, Department of Urology<br>Dinneen, Eoin; University College London Medical School, Department of Surgical and Interventional Sciences<br>Shaw, Greg; University College Hospital London, Department of Urology<br>Carter, Martyn; University College London, Faculty of the Built Environment<br>Patel, Dominic; University College London Cancer Institute, Department of Pathology<br>Moore, Caroline; University College London, Division of Surgery and Interventional Sciences<br>Atkinson, David; University College London, Centre for Medical Imaging<br>Panagiotaki, Eleftheria; University College London, Centre for Medical Imaging Computing, Department of Computer Science<br>Haider, Aiman; University College London Hospitals NHS Foundation Trust, Department of Histopathology<br>Freeman, Alex; University College Hospital London, Department of Histopathology<br>Alexander, Daniel; University College London, Department of Computer Science<br>Punwani, Shonit; University College London Medical School, Centre for

	Medical Imaging
**Primary Subject Heading**:	Radiology and imaging
Secondary Subject Heading:	Urology, Oncology
Keywords:	Magnetic resonance imaging < RADIOLOGY & IMAGING, Prostate disease < UROLOGY, Pathology < NATURAL SCIENCE DISCIPLINES

SCHOLARONE™ Manuscripts

Histo-MRI map study protocol: A prospective cohort study mapping MRI to histology for biomarker validation and prediction of prostate cancer

Saurabh Singh[1*†], Manju Mathew[1,2†], Thomy Mertzanidou[3], Shipra Suman[1,3], Joey Clemente[1], Adam Retter[1], Marianthi-Vasiliki Papoutsaki[1], Lorna Smith[1], Francesco Grussu[3,4], Veeru Kasivisvanathan[5,6] Alistair Grey[6,7], Eoin Dinneen[6], Greg Shaw[6,7], Martyn Carter[8], Dominic Patel[9], Caroline Moore[5,6], David Atkinson[1], Eleftheria Panagiotaki[3], Aiman Haider[2], Alex Freeman[2], Daniel Alexander[3], Shonit Punwani[1]

1. Centre for Medical Imaging, Division of Medicine, University College London, London, UK
2. Department of Pathology, University College London Hospitals NHS Foundation Trust, London, UK
3. Centre for Medical Imaging Computing, Department of Computer Science, University College London, UK
4. Radiomics Group, Vall d'Hebron Institute of Oncology, Vall d'Hebron Barcelona Hospital Campus, Barcelona, Spain
5. Division of Surgery & Interventional Science, University College London, London, UK
6. Department of Urology, University College London Hospitals NHS Foundation Trust, London, UK
7. Department of Urology, Barts Health NHS Foundation Trust, London, UK
8. Faculty of the Built Environment, The Bartlett School of Architecture, University College London, London, UK
9. Department of Pathology, University College London Cancer Institute, London, UK

* Corresponding author. Centre for Medical Imaging, University College London, Charles Bell House, 43-45 Foley Street, London W1W 7TS, UK.

saurabh.singh@nhs.net, Tel +44 0207 679 8156

For peer review only - http://bmjopen.bmj.com/site/about/guidelines.xhtml

† Equal contributors

Abstract

Introduction:

Multiparametric magnetic resonance imaging (mpMRI) is now widely used to risk stratify men with a suspicion of prostate cancer and identify suspicious regions for biopsy. However, the technique has modest specificity and a high false-positive rate, especially in men with mpMRI scored as indeterminate (3/5) or likely (4/5) to have clinically significant cancer (Gleason ≥3+4). Advanced MRI techniques have emerged which seek to improve this characterisation and could predict biopsy results non-invasively. Before these techniques are translated clinically, robust histological and clinical validation is required.

Methods and Analysis:

This study aims to clinically validate two advanced MRI techniques in a prospectively recruited cohort of men suspected of prostate cancer. Histological analysis of men undergoing biopsy or prostatectomy will be used for biological validation of biomarkers derived from VERDICT (Vascular and Extracellular Restricted Diffusion for Cytometry in Tumours) and Luminal Water imaging (LWI). In particular, prostatectomy specimens will be processed using 3-D printed patient-specific moulds to allow for accurate MRI and histology mapping. The index tests will be compared to the histological reference standard to derive false positive rate and true positive rate for men with mpMRI scores which are indeterminate (3/5) or likely (4/5) to have clinically significant prostate cancer (csPCa). Histopathological validation from both biopsy and prostatectomy samples will provide the best ground truth in validating promising MRI techniques which could predict biopsy results and help avoid unnecessary biopsies in men suspected of prostate cancer.

Ethics and Dissemination

For peer review only

Ethical approval was granted by the London – Queen Square Research Ethics Committee (19/LO/1803) on 23rd January 2020. Results from the study will be presented at conferences and submitted to peer-reviewed journals for publication. Results will also be available on ClinicalTrials.gov.

Article Summary

Strengths and limitations of this study

- A prospective cohort of men suspected of prostate cancer referred to two tertiary care centres in the UK representative of the population of interest.
- The reference standard to validate novel imaging biomarkers includes both targeted biopsy and matched whole-mount prostatectomy histology without participants deviating from standard clinical care.
- The patient-specific specimen handling protocol for prostatectomy which was developed at this institution allows for accurate matching of MRI and Histology.
- This protocol will produce a rich imaging and histology dataset that can be used to train machine learning algorithms and validate novel imaging biomarkers.
- Novel imaging biomarkers will not influence clinical decisions, which is ethically sound for novel imaging biomarkers which have not been validated clinically.

For peer review only

Trial registration: Histo-MRI is registered on ClinicalTrials.gov; reference NCT04792138.

Keywords: Prostate cancer, Magnetic Resonance Imaging, VERDICT MRI, Luminal Index Imaging, Prostatectomy, RALP, Machine learning

Word Count: 2252

For peer review only - http://bmjopen.bmj.com/site/about/guidelines.xhtml

1. Introduction

The diagnosis of prostate cancer has been transformed by multiparametric MRI (mpMRI) which has become the first line investigation for men suspected to have prostate cancer in many countries[1]. However, diagnosis still relies on invasive biopsy for confirmation. This diagnostic pathway has two main limitations i) The specificity of mpMRI is modest (as low as 40%) and leads to unnecessary negative biopsies ii) There is a sampling error associated with biopsy as it is can miss abnormalities identified on MRI[2][3].

The poor specificity of mpMRI is due to several factors. Benign diseases such as inflammation and atrophy can mimic tumours or make tumours less conspicuous leading to indeterminate results when assessed by radiologists [4–6]. The mpMRI study is assessed qualitatively rather than quantitatively to determine the presence of clinically significant cancer (csPCa) which causes inter-observer variation and subjectivity. Both these factors can lead to a high false-positive rate in men who undergo biopsy after assessment of their mpMRI. Results from a recent trial at our institution showed that men undergoing biopsy with mpMRI scores of Likert 3 (indeterminate for csPCa) and Likert 4 (likely to have csPCa) had false-positive rates of 85% and 40% respectively [7]. However, when mpMRI is scored as highly likely to have csPCa (Likert 5/5), the percentage of false positives is low at 2% [7]. Therefore, there is a clinical need for biomarkers to better stratify men with Likert 3 or Likert 4 mpMRI scores to reduce false positives without missing men with csPCa.

Advanced MRI techniques designed specifically for the prostate aim to improve cancer detection and characterisation by inferring microstructural information from the whole

For peer review only

prostate non-invasively. Vascular, Extracellular and Restricted Diffusion for Cytometry in Tumours (VERDICT MRI) is a specifically designed diffusion technique based on prostate histology that derives estimates of histological parameters non-invasively[8, 9]. Technical validation and early biological validation have been achieved with results outperforming standard diffusion sequences[10, 11]. Luminal index imaging (LWI) is an advanced T2 based technique that has similarly been technically and biologically validated[12]. Quantitative evaluation of both these techniques could assist radiologists to reduce false positives when mpMRIs are scored as Likert 3 or 4 on the likelihood of csPCa.

Histological validation from biopsy alone has limitations of only validating small regions of the prostate. In contrast, histological validation from prostatectomy specimens leads to a selection bias where men with abnormal prostates are selected rather than those men who may have indolent or benign diseases. Furthermore, the prostate can be sliced in a different axis in histopathology compared to imaging which causes imperfect matching and poor validation. A patient-specific specimen handling for prostatectomy specimens can overcome these limitations and allow better matching of MRI and whole-mount histopathology[13]

In this study, both biopsy and MRI matched whole-mount histology from prostatectomy will be used for clinical validation of novel MRI techniques in a prospective cohort of men suspected of prostate cancer. In particular, the impact of index tests on the false positive rate in men who are scored Likert 3 or 4 and underwent biopsy or prostatectomy will be evaluated. The decision to biopsy or recommendation for prostatectomy will not be influenced by the novel MRI techniques being validated.

For peer review only - http://bmjopen.bmj.com/site/about/guidelines.xhtml

2. Methods and analysis

2.1. Study Design

Histo-MRI is an observational, prospective, cohort study recruiting men with clinical suspicion of prostate cancer from two centres: University College London Hospital (UCLH) and Barts Health. The index tests (VERDICT MRI and LWI) and standard of care mpMRI will be performed at one centralised centre (UCLH). The study opened for recruitment in October 2020 and the anticipated study end date is October 2023. Participants will undergo the both tests before undergoing biopsy if indicated by the standard test. The index tests will not be used to select patients for biopsy. If a recruited participant is diagnosed with prostate cancer and chooses to undergo prostatectomy, the prostate specimen will undergo a specific specimen handling protocol designed to align histopathology to MRI (Figure 1).

The primary objective is to assess whether the index tests can reduce false positives from mpMRI by 20% for men undergoing biopsy or biopsy and prostatectomy. Clinically significant prostate cancer (csPCa) for biopsies is defined as any single biopsy core containing Gleason grade 3+4 or above. For prostatectomy, csPCa is defined as the predominant grade of 3+4 or above in the same matched region on whole mount histology as the abnormality on MRI.

The secondary objectives will examine the proportions of true positive lesions from the index tests.

For peer review only

Exploratory analyses will determine the correlation of VERDICT and LWI parameters to histological parameters. For VERDCIT MRI: fractional intracellular fraction (FIC) to fractional histological intracellular component, fractional extracellular extravascular space (FEES) to histological glandular and stromal component and fractional vascular volume (FVASC) to histological vascular component. For LWI, luminal water fraction (LWF) will be correlated to luminal space fraction.

Matched MR and histology images will be used as training data for machine learning algorithms. The algorithms will be used to solve a variety of regression problems where the input is MRI and the output is the histological features in each voxel or the full histological image appearance itself, building on previous work from our group[14–16].

2.2. Participants

Participants will be approached consecutively from Urology clinics at the point of referral from two centres: University College London Hospital Foundation Trust, London, UK and Barts Health, London, UK. Inclusion and exclusion criteria are stated in Table 1. Informed Consent will be obtained on the day of the index test and participants will be given at least 24 hours before being consented to consider participation.

2.3. Index tests

The index tests of VERDICT MRI and LWI will be performed on a 3T MRI scanner (Achieva or Ingenia, Philips Healthcare, Best, Netherlands). Sequence parameters are detailed in Table 2 and 3. These will be additional to the clinical multiparametric sequence. Total scan time will be a maximum of 1 hour.

For peer review only - http://bmjopen.bmj.com/site/about/guidelines.xhtml

The VERDICT MRI technique has been described in previous publications[11]; a summary is given below. VERDICT uses a pulse-gradient spin-echo sequence acquired with a 32-channel cardiac coil with b values of 90-3000 s/mm^2 in 3 orthogonal directions. For b=90 s/mm^2, the number of signal averages (NSA) was 4 and for other b values, the NSA was 6. The voxel size is 1.3mm x 1.3mm x 5 mm, matrix size =176 x 176. A b=0 s/mm^2 image for every echo time (TE) is acquired to mitigate T2 dependence. After processing, estimates of intracellular volume fraction (FIC), extravascular extracellular volume fraction (EES), vascular volume fraction (FVASC) are generated by a previously described method[17].

LWI comprises of a multi-echo spin-echo sequence with an echo spacing of 31.25 msec and repetition time (TR) of 8956 msec. The field of view (FOV) is 180 × 180 mm and acquired voxel size = 0.9 mm × 0.9 mm × 3.5 mm with a scan duration of 5 minutes 50 seconds. LWF values for lesions identified on mpMRI will be calculated after data processing by a previously described method[7].

2.4. Data analysis

Reporting of clinical mpMRI will follow standard of care and be reported by Uro-radiologists based at UCLH on an ordinal Likert scale (1 to 5): 1- tumour highly unlikely, 2- tumour unlikely, 3- equivocal, 4- tumour likely and 5- tumour highly likely. If the mpMRI study is reported as Likert 3 or 4 and the patient is offered a biopsy, a radiologist (blinded to biopsy results/histopathology) will use the pictorial report to derive index test quantitative parameters: FIC for VERDICT MRI and LWF from LWI. Thresholds of index test parameters based on previous work from the INNOVATE trial

For peer review only - http://bmjopen.bmj.com/site/about/guidelines.xhtml

will be applied to determine whether a lesion is positive or negative[7]. This will be compared to a histological reference standard allowing assessment of false positive rate, sensitivity and specificity.

2.5. Reference Standard

The reference standard for those men who undergo biopsy and/or prostatectomy will be histopathological diagnosis (Figure 2). Abnormal regions identified by clinical mpMRI will be targeted at biopsy. Men who elect to have prostatectomy after positive biopsy will be processed by a published specimen handling protocol developed at our institution[13]. Histology from biopsy and prostatectomy specimens will be assessed by two histopathologists blinded to MRI findings. Gleason grade for targeted biopsy and matched prostatectomy whole block section will be analysed and ascribed a Gleason score on consensus. Assessors of the reference standard will be blinded to MRI results. If there is a disagreement in histological assessment for a participant who undergoes both targeted biopsy and prostatectomy, the prostatectomy lesion grading will supersede.

For patients that undergo prostatectomy, in order to match MRI slices to histopathology, a patient specific specimen handling protocol is to be used[13] (Figure 1). This protocol is needed because in standard histological processing there are changes in orientation after cutting and sampling which are difficult to account for when matching standard whole mount histology to MRI (Figure 1). We create a personalised 3-D printed mould for men undergoing prostatectomy using their pre-operative MRI images (T2W imaging) in order to allow for predictable sectioning.

For peer review only - http://bmjopen.bmj.com/site/about/guidelines.xhtml

For peer review only

After surgical removal of the prostate the specimen will be positioned in the mould and scanned in a 3T MRI scanner. This will facilitate the matching between pre-operative MRI and histology.

The mould defines a reference slice which has two cutting planes spaced 5 mm apart. A twin bladed knife is used to cut a 5 mm slice pre-defined from the mould. The rest of the prostate is then sliced at 5 mm thickness as per standard laboratory protocol. Whole mount sections are then processed for haematoxylin and eosin staining. Additional immunohistochemical staining for vascular and stromal structures will be performed to aide in mapping with the index tests.

The matched whole mount histology slice will be assessed and used to determine whether an MRI lesion was positive (≥ Gleason 3+4) or negative (≤Gleason 3+3 or negative).

2.6. Statistical Analyses

A sample size of 128 subjects achieves a 90% power to detect a difference of 20% in the proportion of false positives between the index tests (0.45) and standard test (0.65). This calculation uses a two-sided Pearson Chi-Square test with a significance level of 0.05 and confidence level of 95%. We anticipate a biopsy rate of 57% in the cohort and of those biopsied, 75% to be scored Likert 3 or 4, based on results from the recent INNOVATE trial at our institution[7]. Therefore, a sample size of 300 will provide sufficient power, with an estimated 171 men predicted to have biopsy and of these men, 128 to have a Likert score of 3 or 4.

For peer review only

Approximately 800 patients undergo prostatectomy per year at UCLH and 150 are referred from Barts Health NHS trust. We estimate in a sample size of 300, approximately 50 patients will elect to have a prostatectomy based on clinical experience and recruitment in other studies carried out at our institution.

The difference in proportion of false positives and true positives for the index test will be compared to the standard test (mpMRI) using a Chi-square test. Correlation coefficients will be used to determine the correlation between index test parameters and histological measures.

2.7. Patient and public involvement

There has been no formal involvement of the patient group or public in the design of this protocol. However, participant feedback from recent research studies such as INNOVATE[7] has informed the study design. For instance, participants will be offered research scans on the same appointment date as their hospital appointment for convenience.

3. Discussion

Histology remains the gold standard of prostate cancer diagnosis and therefore represents the best reference standard for novel MRI techniques. This observational study aims to test the predictive capabilities of novel MRI techniques without compromising on standard clinical care. Furthermore, matched MRI and histological data will provide a rich data set for training machine learning algorithms.

For peer review only

For peer review only - http://bmjopen.bmj.com/site/about/guidelines.xhtml

This study design has some limitations. Biopsy decisions will not be influenced by the index tests therefore we cannot determine their true sensitivity and specificity. However, at this stage of biomarker development, prospective validation of thresholds derived from a previous study is required before biopsy decisions could be determined by the index tests [7]. Given the high negative predictive value of mpMRI[18, 19], it would also lead to unnecessary morbidity if patients with negative mpMRI are biopsied. The inherent sampling error with biopsies is also a limitation of this study in men who do not undergo prostatectomy. However, if only men undergoing prostatectomy were selected in this study, histological validation will be limited by spectrum bias where more aggressive tumours are selected.

The results of this study may not be generalisable to other centres. The two index tests have been optimised on scanners from the same vendor. Formal reproducibility studies are required to assess whether the index tests perform as well on different systems before multi-centre trials can be performed[20].

Conclusion

Histo-MRI is a prospective, observational study, which aims to test the potential value of novel MRI techniques in diagnosis of significant prostate cancer in men that undergo biopsy following mpMRI. The results of this study will provide histological validation for novel MRI techniques and produce a rich dataset which can be used to train machine learning algorithms for prostate cancer diagnosis and prognosis.

4. Ethics and Dissemination

For peer review only

The study is sponsored by University College London. The UCL/UCLH joint research office maintains responsibility for monitoring of Good Clinical Practice in the study. Ethical approval for the study was granted by the London – Queen Square Research Ethics Committee (19/LO/1803) on 23rd January 2020. Study results will be presented at conferences and submitted to peer reviewed journals.

List of abbreviations

MpMRI – multiparametric magnetic resonance imaging

VERDICT - Vascular and Extracellular Restricted Diffusion for Cytometry in Tumours

LWI – Luminal Index imaging

UCLH – University College London Hospital

UCL – University College London

FIC – Fractional Intracellular Volume

FEES – Fractional Extracellular extravascular space

FVASC – Fractional vascular space

LWF – Luminal water fraction

csPCa – Clinically significant prostate cancer

TE – Echo time

TR – Time to repetition

FOV – Field of view

For peer review only

- Contribution statement
    - Study concept and initial design: DA, SP, TM, EP
    - Study design and statistical analysis: SS, FG, TM, AH, AF, DA, SP
    - Data acquisition and analysis: SS, MM, TM, ShS, JC, AR, VP, LS, VK, AG, ED, GS, MC, DP, CM, EP, AH, AF.
    - All authors read and approved the final manuscript.
- Competing interests statement
    - The work of Baris Kanber, Shonit Punwani and Daniel Alexander is supported by the National Institute for Health Research University College London Hospitals Biomedical Research Centre.
    - Veeru Kasivisvanathan is an Academic Clinical Lecturer funded by the United Kingdom National Institute for Health Research (NIHR). The views expressed in this publication are those of the author(s) and not necessarily those of the NHS, the National Institute for Health Research or the Department of Health.
    - CMM is supported by an NIHR Research Professorship, and has additional study funding from the Medical Research Council, Cancer Research UK, PCUK, Movember, and the European Association of Urology Research Board.

For peer review only

- FG is currently supported by PREdICT, an investigator-initiated study at the Vall d'Hebron Hospital campus (Barcelona, Spain), funded by AstraZeneca.

- Funding statement
  - This work is supported by Engineering and Physical Sciences Research Council (EPSRC), grant reference (EP/R006032/1) and EP/M020533/1.

- Data Sharing Statement
  - All data relevant to the study are included in the article.

REFERENCES

1. Recommendations | Prostate cancer: diagnosis and management | Guidance | NICE.

2. Ahmed HU, El-Shater Bosaily A, Brown LC, Gabe R, Kaplan R, Parmar MK, et al. Diagnostic accuracy of multi-parametric MRI and TRUS biopsy in prostate cancer (PROMIS): a paired validating confirmatory study. Lancet. 2017;389:815–22.

3. Kasivisvanathan V, Rannikko AS, Borghi M, Panebianco V, Mynderse LA, Vaarala MH, et al. MRI-targeted or standard biopsy for prostate-cancer diagnosis. N Engl J Med. 2018;378:1767–77.

4. Kitzing YX, Prando A, Varol C, Karczmar GS, Maclean F, Oto A. Benign conditions that mimic prostate carcinoma: MR imaging features with histopathologic correlation. Radiographics. 2016;36:162–75. doi:10.1148/rg.2016150030.

5. Osses DF, Arsov C, Schimmöller L, Schoots IG, van Leenders GJLH, Esposito I, et al. Equivocal pi-rads three lesions on prostate magnetic resonance imaging: Risk stratification strategies to avoid mri-targeted biopsies. J Pers Med. 2020;10:1–13. doi:10.3390/jpm10040270.

6. Schoots IG. MRI in early prostate cancer detection: How to manage indeterminate or equivocal PI-RADS 3 lesions? Translational Andrology and Urology. 2018;7:70–82.

For peer review only - http://bmjopen.bmj.com/site/about/guidelines.xhtml

doi:10.21037/tau.2017.12.31.

7. Pye H, Singh S, Norris JM, Carmona Echeverria LM, Stavrinides V, Grey A, et al. Evaluation of PSA and PSA Density in a Multiparametric Magnetic Resonance Imaging-Directed Diagnostic Pathway for Suspected Prostate Cancer: The INNOVATE Trial. Cancers (Basel). 2021;13:1985. doi:10.3390/cancers13081985.

8. Panagiotaki E, Chan RW, Dikaios N, Ahmed HU, O'Callaghan J, Freeman A, et al. Microstructural characterization of normal and malignant human prostate tissue with vascular, extracellular, and restricted diffusion for cytometry in tumours magnetic resonance imaging. Invest Radiol. 2015;50:218–27.

9. Panagiotaki E, Walker-Samuel S, Siow B, Johnson SP, Rajkumar V, Pedley RB, et al. Noninvasive quantification of solid tumor microstructure using VERDICT MRI. Cancer Res. 2014;74:1902–12. doi:10.1158/0008-5472.CAN-13-2511.

10. Bailey C, Bourne RM, Siow B, Johnston EW, Brizmohun Appayya M, Pye H, et al. VERDICT MRI validation in fresh and fixed prostate specimens using patient-specific moulds for histological and MR alignment. NMR Biomed. 2019;32.

11. Johnston EW, Bonet-Carne E, Ferizi U, Yvernault B, Pye H, Patel D, et al. VERDICT MRI for prostate cancer: Intracellular volume fraction versus apparent diffusion coefficient. Radiology. 2019;291:391–7.

12. Devine W, Giganti F, Johnston EW, Sidhu HS, Panagiotaki E, Punwani S, et al. Simplified Luminal Water Imaging for the Detection of Prostate Cancer From Multiecho T_2 MR Images. J Magn Reson Imaging. 2019;50:910–7. doi:10.1002/jmri.26608.

13. Bourne RM, Bailey C, Johnston EW, Pye H, Heavey S, Whitaker H, et al. Apparatus for histological validation of in vivo and ex vivo magnetic resonance imaging of the human prostate. Front Oncol. 2017;7 MAR:47. doi:10.3389/fonc.2017.00047.

14. Jacobs JG, Panagiotaki E, Alexander DC. Gleason grading of prostate tumours with max-margin conditional random fields. Lect Notes Comput Sci (including Subser Lect

For peer review only

Notes Artif Intell Lect Notes Bioinformatics). 2014;8679:85–92. doi:10.1007/978-3-319-10581-9_11.

15. Tanno R, Worrall DE, Ghosh A, Kaden E, Sotiropoulos SN, Criminisi A, et al. Bayesian Image Quality Transfer with CNNs: Exploring Uncertainty in dMRI Super-Resolution. Lect Notes Comput Sci (including Subser Lect Notes Artif Intell Lect Notes Bioinformatics). 2017;10433 LNCS:611–9. http://arxiv.org/abs/1705.00664. Accessed 11 Feb 2021.

16. Alexander DC, Zikic D, Zhang J, Zhang H, Criminisi A. Image quality transfer via random forest regression: Applications in diffusion MRI. In: Lecture Notes in Computer Science (including subseries Lecture Notes in Artificial Intelligence and Lecture Notes in Bioinformatics). Springer Verlag; 2014. p. 225–32. doi:10.1007/978-3-319-10443-0_29.

17. Panagiotaki E, Walker-Samuel S, Siow B, Johnson SP, Rajkumar V, Pedley RB, et al. Noninvasive quantification of solid tumor microstructure using VERDICT MRI. Cancer Res. 2014;74:1902–12. doi:10.1158/0008-5472.CAN-13-2511.

18. Sathianathen NJ, Omer A, Harriss E, Davies L, Kasivisvanathan V, Punwani S, et al. Negative Predictive Value of Multiparametric Magnetic Resonance Imaging in the Detection of Clinically Significant Prostate Cancer in the Prostate Imaging Reporting and Data System Era: A Systematic Review and Meta-analysis. European Urology. 2020;78:402–14. doi:10.1016/j.eururo.2020.03.048.

19. Itatani R, Namimoto T, Atsuji S, Katahira K, Morishita S, Kitani K, et al. Negative predictive value of multiparametric MRI for prostate cancer detection: outcome of 5-year follow-up in men with negative findings on initial MRI studies. Eur J Radiol. 2014;83:1740–5. doi:10.1016/j.ejrad.2014.06.026.

20. O'Connor JPB, Aboagye EO, Adams JE, Aerts HJWL, Barrington SF, Beer AJ, et al. Imaging biomarker roadmap for cancer studies. Nat Rev Clin Oncol. 2017;14:169–86. doi:10.1038/nrclinonc.2016.162.

For peer review only

Figure Legends

Figure 1 Protocol for matching MRI to Whole mount histology.

The participant's prostate is contoured by a radiologist on pre-operative imaging ('in-vivo MRI') slice by slice. Based on these contours a mould specific to the participant's prostate is 3-D printed. The 'reference slice' is pre-defined based on the location of the tumour. After prostatectomy, the prostate is scanned in the mould ('ex-vivo MRI'). The prostate is sectioned first at the pre-defined reference slice. The remainder of the prostate is then sliced as standard. Stained 'whole mount histology' is then matched with ex-vivo and in-vivo imaging.

Figure 2 Reference standard flow chart

Reference standard derived from multi-parametric (mpMRI) and histology for index tests. Histology refers to either a positive biopsy core in a targeted lesion or positive lesion on matched MRI and whole mount histology from prostatectomy. Histology from prostatectomy supersedes targeted biopsy.

For peer review only

Table 1 – Inclusion, exclusion and withdrawal criteria

**Patient Inclusion Criteria**
Biopsy naïve men with clinical suspicion of prostate cancer
**Patient Exclusion Criteria**
Men unable to have an MRI scan or in whom artifact would reduce quality of MRI
Men unable to given informed consent
Previous treatment of prostate cancer (surgery, radiotherapy, hormone treatment)
Previous biopsy
Withdrawal Criteria
Images inadequate for analysis

For peer review only

Table 2 – Sequence parameters for VERDICT MRI

VERDICT MRI		
**MR scanner**	**Achieva (3T)**	**Ingenia (3T)**
Receive coil (s)	32 channel Cardiac coil	Body coils
Sequence	DWI SE EPI single shot	DWI SE EPI single shot
Field of View (mm)	220	220
Number of slices	14	14
Slice thickness (mm)	5	5
Slice gap (mm)	0	0
phase encoding direction	AP	AP
Reconstructed matrix	176 x 176	176 x 176
Reconstructed pixel size (mm)	1.25	1.25
**b-values**	0,3000	0,3000
Repetition time (TR) range, actual (ms)	3349-10000,2260	3349-10000, 6292
Echo time (TE) (ms)	80	87
Water fat shift WFS(pix)/Bandwidth(Hz)	49.09/8.8	57.54/7.5
DELTA/delta (ms)	38.8/18.9	43.4/20.0
Number of signal averages	6	6
**b-values**	0, 2000	0, 2000
TR range, actual (ms)	2000-10000, 3897	2000-10000, 6699
TE(ms)	67	75
WFS(pix)/BW(Hz)	49.09/8.8	57.55,7.5
DELTA/delta (ms)	32.3/12.4	37.4, 14.0
Number of signal averages	6	6
**b-values**	0,1500	0, 1500
TR range, actual (ms)	2000-10000, 2398	2000-10000, 2967
TE(ms)	90	94
WFS(pix)/BW(Hz)	49.09/8.8	58.05, 7.5
DELTA/delta (ms)	43.8/23.9	46.9, 23.3
Number of signal averages	6	6
**b-values**	0, 500	0, 500
TR range, actual (ms)	2482-10000, 2482	2000-10000, 2229
TE(ms)	65	68
WFS(pix)/BW(Hz)	49.06/8.8	58.05, 7.5
DELTA/delta (ms)	31.3, 11.4	33.9, 10.3
Number of signal averages	6	6
**b-values**	0, 90	0, 90
TR range, actual (ms)	2482-10000, 2482	2000-10000, 2024
TE(ms)	50	54
WFS(pix)/BW(Hz)	49.09,8.8	57.54, 7.5
DELTA/delta (ms)	23.8/3.9	26.9, 3.5
Number of signal averages	4	4
Acquisition Time (minutes: seconds)	10:95	17:41

For peer review only

For peer review only - http://bmjopen.bmj.com/site/about/guidelines.xhtml

Table 3 – Sequence parameters for Luminal Index Imaging MRI

Luminal Index Imaging		
**MR scanner**	**Achieva**	**Ingenia**
Receive coil (s)	32 channel Cardiac coil	Body Coils
**Sequence**	TSE (multishot)	FSE
**FOV (mm)**	180	180
Number of slices	19	19
**Slice thickness (mm)**	3.5	3.5
**Slice gap (mm)**	0.35	0.35
Phase Encoding direction	Right Left	Right Left
Reconstructed matrix (read)	224 x 224	224 x 224
Reconstructed pixel size (mm x mm)	0.94 x 0.94	0.94 x 0.94
Echo times (ms)	31.25/ 62.5/ 93.8/ 125/ 156.3/ 187.5/ 218.8/ 250	31.25/ 62.5/ 93.8/ 125/ 156.3/ 187.5/ 218.8/ 250
Repetition time (ms)	Shortest (7676ms)	Shortest (7676ms)
Number of echoes	8	8
Receive bandwidth	WFS 2.99 / (144.8.6Hz/px)	WFS 2.99 / (144.8.6Hz/px)
Number of signal averages	1	1
Turbo factor	8	8
Acquisition time (minutes: seconds)	06:44	05:39

For peer review only

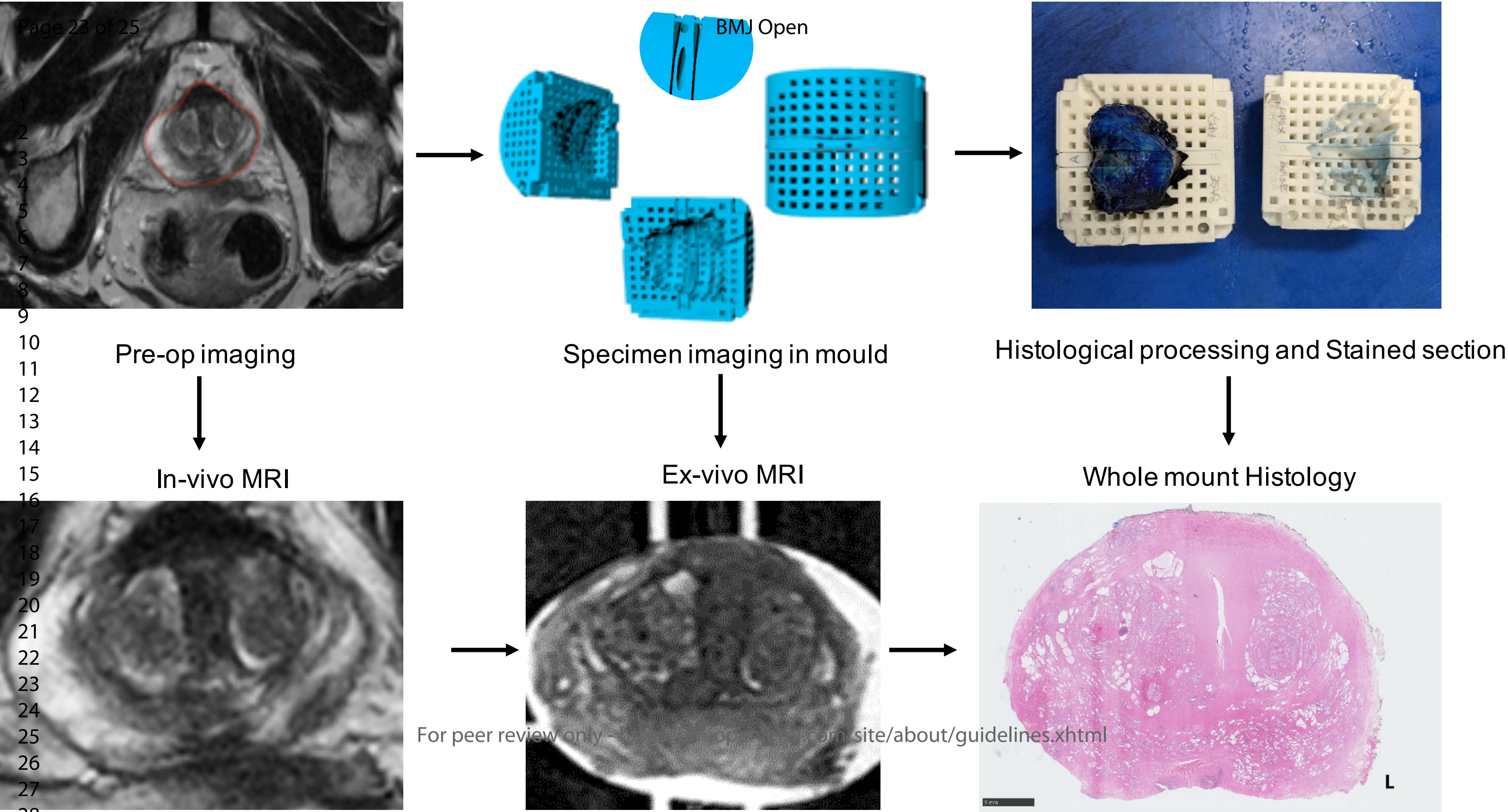

For peer review only - [illegible].com/site/about/guidelines.xhtml

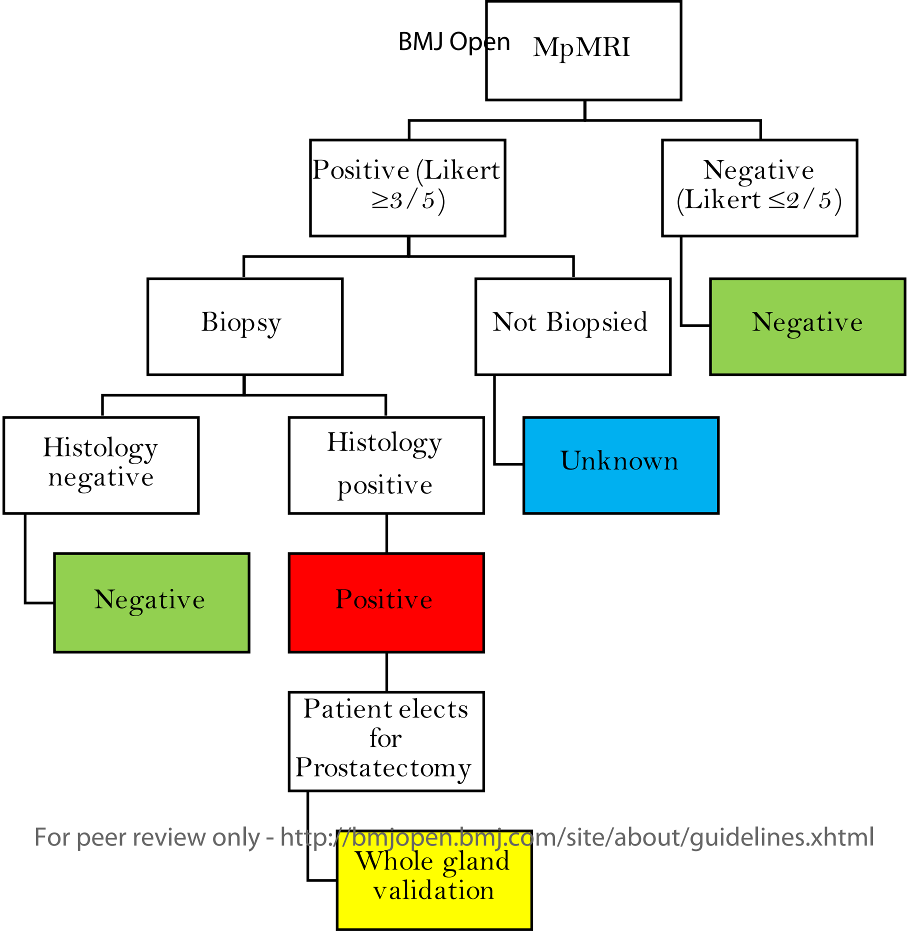
MpMRI
Positive (Likert ≥3/5)
Negative (Likert ≤2/5)
Biopsy
Not Biopsied
Negative
Histology negative
Histology positive
Unknown
Negative
Positive
Patient elects for Prostatectomy
Whole gland validation

Section & Topic	No	Item	Reported on page #
**TITLE OR ABSTRACT**			
	**1**	Identification as a study of diagnostic accuracy using at least one measure of accuracy (such as sensitivity, specificity, predictive values, or AUC)	1
**ABSTRACT**			
	**2**	Structured summary of study design, methods, results, and conclusions (for specific guidance, see STARD for Abstracts)	2
**INTRODUCTION**			
	**3**	Scientific and clinical background, including the intended use and clinical role of the index test	4
	**4**	Study objectives and hypotheses	4
**METHODS**			
Study design	**5**	Whether data collection was planned before the index test and reference standard were performed (prospective study) or after (retrospective study)	6
Participants	**6**	Eligibility criteria	7
	**7**	On what basis potentially eligible participants were identified (such as symptoms, results from previous tests, inclusion in registry)	7
	**8**	Where and when potentially eligible participants were identified (setting, location and dates)	7
	**9**	Whether participants formed a consecutive, random or convenience series	7
Test methods	**10a**	Index test, in sufficient detail to allow replication	6
	**10b**	Reference standard, in sufficient detail to allow replication	7, 8
	**11**	Rationale for choosing the reference standard (if alternatives exist)	7
	**12a**	Definition of and rationale for test positivity cut-offs or result categories of the index test, distinguishing pre-specified from exploratory	7, 8
	**12b**	Definition of and rationale for test positivity cut-offs or result categories of the reference standard, distinguishing pre-specified from exploratory	7, 8
	**13a**	Whether clinical information and reference standard results were available to the performers/readers of the index test	7, 8
	**13b**	Whether clinical information and index test results were available to the assessors of the reference standard	7, 8
Analysis	**14**	Methods for estimating or comparing measures of diagnostic accuracy	8
	**15**	How indeterminate index test or reference standard results were handled	8
	**16**	How missing data on the index test and reference standard were handled	8
	**17**	Any analyses of variability in diagnostic accuracy, distinguishing pre-specified from exploratory	9, 10
	**18**	Intended sample size and how it was determined	10
**RESULTS**			
Participants	**19**	Flow of participants, using a diagram	N/A
	**20**	Baseline demographic and clinical characteristics of participants	N/A
	**21a**	Distribution of severity of disease in those with the target condition	N/A
	**21b**	Distribution of alternative diagnoses in those without the target condition	N/A
	**22**	Time interval and any clinical interventions between index test and reference standard	N/A
Test results	**23**	Cross tabulation of the index test results (or their distribution) by the results of the reference standard	N/A
	**24**	Estimates of diagnostic accuracy and their precision (such as 95% confidence intervals)	N/A
	**25**	Any adverse events from performing the index test or the reference standard	N/A
**DISCUSSION**			
	**26**	Study limitations, including sources of potential bias, statistical uncertainty, and generalisability	11
	**27**	Implications for practice, including the intended use and clinical role of the index test	NA
**OTHER INFORMATION**			
	**28**	Registration number and name of registry	3
	**29**	Where the full study protocol can be accessed	3
	**30**	Sources of funding and other support; role of funders	21

For peer review only - http://bmjopen.bmj.com/site/about/guidelines.xhtml

For peer review only

cc BY SA

STARD 2015

AIM

STARD stands for "Standards for Reporting Diagnostic accuracy studies". This list of items was developed to contribute to the completeness and transparency of reporting of diagnostic accuracy studies. Authors can use the list to write informative study reports. Editors and peer-reviewers can use it to evaluate whether the information has been included in manuscripts submitted for publication.

EXPLANATION

A **diagnostic accuracy study** evaluates the ability of one or more medical tests to correctly classify study participants as having a **target condition.** This can be a disease, a disease stage, response or benefit from therapy, or an event or condition in the future. A medical test can be an imaging procedure, a laboratory test, elements from history and physical examination, a combination of these, or any other method for collecting information about the current health status of a patient.

The test whose accuracy is evaluated is called **index test.** A study can evaluate the accuracy of one or more index tests. Evaluating the ability of a medical test to correctly classify patients is typically done by comparing the distribution of the index test results with those of the **reference standard**. The reference standard is the best available method for establishing the presence or absence of the target condition. An accuracy study can rely on one or more reference standards.

If test results are categorized as either positive or negative, the cross tabulation of the index test results against those of the reference standard can be used to estimate the **sensitivity** of the index test (the proportion of participants *with* the target condition who have a positive index test), and its **specificity** (the proportion *without* the target condition who have a negative index test). From this cross tabulation (sometimes referred to as the contingency or "2x2" table), several other accuracy statistics can be estimated, such as the positive and negative **predictive values** of the test. Confidence intervals around estimates of accuracy can then be calculated to quantify the statistical **precision** of the measurements.

If the index test results can take more than two values, categorization of test results as positive or negative requires a **test positivity cut-off**. When multiple such cut-offs can be defined, authors can report a receiver operating characteristic (ROC) curve which graphically represents the combination of sensitivity and specificity for each possible test positivity cut-off. The **area under the ROC curve** informs in a single numerical value about the overall diagnostic accuracy of the index test.

The **intended use** of a medical test can be diagnosis, screening, staging, monitoring, surveillance, prediction or prognosis. The **clinical role** of a test explains its position relative to existing tests in the clinical pathway. A replacement test, for example, replaces an existing test. A triage test is used before an existing test; an add-on test is used after an existing test.

Besides diagnostic accuracy, several other outcomes and statistics may be relevant in the evaluation of medical tests. Medical tests can also be used to classify patients for purposes other than diagnosis, such as staging or prognosis. The STARD list was not explicitly developed for these other outcomes, statistics, and study types, although most STARD items would still apply.

DEVELOPMENT

This STARD list was released in 2015. The 30 items were identified by an international expert group of methodologists, researchers, and editors. The guiding principle in the development of STARD was to select items that, when reported, would help readers to judge the potential for bias in the study, to appraise the applicability of the study findings and the validity of conclusions and recommendations. The list represents an update of the first version, which was published in 2003.

More information can be found on http://www.equator-network.org/reporting-guidelines/stard.

For peer review only
