# Supplementary material for: Histo-MRI map study protocol: a prospective cohort study mapping MRI to histology for biomarker validation and prediction of prostate cancer
Source: BMJ Open. 2022 Apr 8;12(4):e059847. doi: 10.1136/bmjopen-2021-059847 (PMC8995953; doi:10.1136/bmjopen-2021-059847)
Supplement: Reviewer comments [file bmjopen-2021-059847.reviewer_comments.pdf]

PEER REVIEW HISTORY

BMJ Open publishes all reviews undertaken for accepted manuscripts. Reviewers are asked to complete a checklist review form (**http://bmjopen.bmj.com/site/about/resources/checklist.pdf**) and are provided with free text boxes to elaborate on their assessment. These free text comments are reproduced below.

ARTICLE DETAILS

**TITLE (PROVISIONAL)**	Histo-MRI map study protocol: A prospective cohort study mapping MRI to histology for biomarker validation and prediction of prostate cancer
**AUTHORS**	Singh, Saurabh; Mathew, Manju; Mertzanidou, Thomy; Suman, Shipra; Clemente, Joey; Retter, Adam; Papoutsaki, Marianthi-Vasiliki; Smith, Lorna; Grussu, Francesco; Kasivisvanathan, Veeru; Grey, Alistair; Dinneen, Eoin; Shaw, Greg; Carter, Martyn; Patel, Dominic; Moore, Caroline; Atkinson, David; Panagiotaki, Eleftheria; Haider, Aiman; Freeman, Alex; Alexander, Daniel; Punwani, Shonit

This article was not externally reviewed at BMJ Open. Protocols that have been independently assessed prior to submission to BMJ Open are usually fast-tracked to publication on the grounds that further substantial changes will not be possible. This independent assessment will usually be external, independent review for both a substantial grant award from a non-commercial or government funder and ethics approval.
